# Formate and hydrogen in hydrothermal vents and their use by extremely thermophilic methanogens and heterotrophs

**DOI:** 10.3389/fmicb.2023.1093018

**Published:** 2023-03-06

**Authors:** James F. Holden, Harita Sistu

**Affiliations:** Department of Microbiology, University of Massachusetts, Amherst, MA, United States

**Keywords:** formate, hydrogen, hydrothermal vent, hyperthermophiles, formate dehydrogenase, hydrogenase, *Thermococci*, *Methanococci*

## Abstract

Extremely thermophilic methanogens in the *Methanococci* and heterotrophs in the *Thermococci* are common in deep-sea hydrothermal vents. All *Methanococci* use H_2_ as an electron donor, and a few species can also use formate. Most *Methanococci* have a coenzyme F_420_-reducing formate dehydrogenase. All *Thermococci* reduce S^0^ but have hydrogenases and produce H_2_ in the absence of S^0^. Some *Thermococci* have formate hydrogenlyase (Fhl) that reversibly converts H_2_ and CO_2_ to formate or an NAD(P)^+^-reducing formate dehydrogenase (Nfd). Questions remain if *Methanococci* or *Thermococci* use or produce formate in nature, why only certain species can grow on or produce formate, and what the physiological role of formate is? Formate forms abiotically in hydrothermal fluids through chemical equilibrium with primarily H_2_, CO_2_, and CO and is strongly dependent upon H_2_ concentration, pH, and temperature. Formate concentrations are highest in hydrothermal fluids where H_2_ concentrations are also high, such as in ultramafic systems where serpentinization reactions occur. In nature, *Methanococci* are likely to use formate as an electron donor when H_2_ is limiting. *Thermococci* with Fhl likely convert H_2_ and CO_2_ to formate when H_2_ concentrations become inhibitory for growth. They are unlikely to grow on formate in nature unless formate is more abundant than H_2_ in the environment. Nearly all *Methanococci* and *Thermococci* have a gene for at least one formate dehydrogenase catalytic subunit, which may be used to provide free formate for *de novo* purine biosynthesis. However, only species with a membrane-bound formate transporter can grow on or secrete formate. Interspecies H_2_ transfer occurs between *Thermococci* and *Methanococci*. This and putative interspecies formate transfer may support *Methanococci* in low H_2_ environments, which in turn may prevent growth inhibition of *Thermococci* by its own H_2_. Future research directions include understanding when, where, and how formate is used and produced by these organisms in nature, and how transcription of *Thermococci* genes encoding formate-related enzymes are regulated.

## 1. Introduction

It was estimated that 40% of bacterial and archaeal global biomass is found in the rocky portion of the ocean crust below ocean sediments (Bar-On et al., 2018; Fleming and Wuertz, 2019). These microbes live in cracks and pores of the rocky subseafloor in the absence of sunlight and often in the absence of oxygen and rely on the gases, aqueous compounds (e.g., sulfide, sulfate, and nitrate), organic compounds, and minerals found locally for growth. In high-temperature anoxic environments, H_2_ is generally considered to be the primary electron donor and CO_2_ the primary carbon source for autotrophic metabolism. However, recently other electron donors and carbon sources such as formate have been considered as alternatives (Windman et al., 2007), especially in high pH environments where dissolved inorganic carbon precipitates as calcium carbonate and is largely unavailable to autotrophs (Lang et al., 2018; McGonigle et al., 2020; Brazelton et al., 2022). There are strong links between formate and H_2_ in hydrothermal environments and in the physiology of microbes that consume and produce formate and H_2_.

High-temperature microbes that use formate and H_2_ are examined herein, namely methanogens (in the class *Methanococci* and the class *Methanopyri*) and heterotrophs (in the class *Thermococci*). These organisms are found in deep-sea hydrothermal vents on or near tectonic plate boundaries – both mid-ocean ridges and subduction zones. Thermophiles and hyperthermophiles are defined as those organisms with optimal growth temperatures above 50°C and 80°C, respectively (Stetter, 2006). In this review, the term ‘extreme thermophile’ will be used to describe organisms with optimal growth temperatures above 65°C. Extremely thermophilic *Methanococci* and *Thermococci* are among the more cosmopolitan and well-studied microbes found in hydrothermal vent environments. All *Methanococci* and the marine hyperthermophile *Methanopyrus kandleri* (the sole member of the *Methanopyri*) use H_2_ and CO_2_ as energy and carbon sources to produce CH_4_, H_2_O, and biomass (Thauer et al., 2008). All *Thermococci* use peptides and sugars as carbon and energy sources and reduce zero-valent sulfur (S^0^) to a sulfide species or reduce 2 H^+^ to H_2_ in the absence of S^0^ (Wu et al., 2018). However, some extremely thermophilic *Methanococci* and *Thermococci* grow using formate as an energy source only or as both energy and carbon sources (Belay et al., 1986; Kim et al., 2010; Lim et al., 2014). This raises questions about which organisms can use formate, when they use formate in nature, and for what purpose. This review describes how formate and H_2_ are formed in hydrothermal vents, the concentrations of these compounds in pure hydrothermal fluids, the physiology of extremely thermophilic *Methanococci* and *Thermococci* as it relates to formate and H_2_ use, transcriptional regulation of formate dehydrogenase and hydrogenase genes, and suggests likely roles for formate use by these organisms in nature.

## 2. Abiotic H_2_ production in hydrothermal vents

**FIGURE 1:**
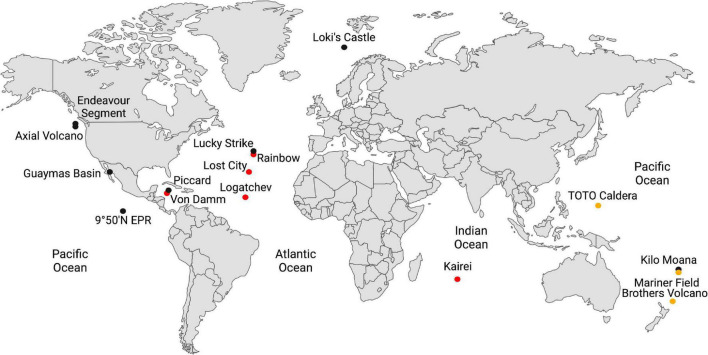
Global map of the hydrothermal vents in Table 1. Hydrothermal vents hosted in ultramafic rock are shown in red; basalt, in black; and in subduction zones, in yellow-brown.

Deep-sea hydrothermal vents provide one of the best access points to the hydrothermally influenced portion of the rocky subseafloor and are ideal starting points for understanding biogeochemical processes in these regions of the crust. Some hydrothermal fluids rise through the crust undiluted, so-called “end-member hydrothermal fluid,” and exit the seafloor at temperatures generally above 300°C (Table 1). It can also mix with cold seawater on or below the seafloor creating habitats for extremely thermophilic anaerobes either within the host rock (e.g., basalt) or in metal sulfide mineral precipitates (e.g., black smoker chimneys). Most hydrothermal vent studies are focused on one of three types of sites: ultramafic sites along slow-to-ultraslow tectonic spreading centers, mafic sites along intermediate-to-fast spreading centers, and subduction-influenced sites near tectonic convergence zones (Figure 1).

**TABLE 1:** Physical, chemical, and microbial characteristics of hydrothermal vent sites.

Location	T_max_ (°C)	pH	H_2_ (mM)*^a^*	Formate (μM)*^a^*	*Methanococci*	*Thermococci*
**Ultramafic (peridotite)-influenced sites**						
Kairei*^b^*	365	3.4–3.6	2.5–8.2	–	M	M
Logatchev*^c^*	350	6.2	5.9	–	F	ND
Lost City*^d^*	90	9.5–10.9	1.2–15.1	36–158	ND	M
Rainbow*^e^*	370	3.0–3.4	12.3–16.9	–	M	M
Von Damm*^f^*	226	5.6–6.1	9.9–18.3	82–669	F	F
**Mafic (basalt-hosted) sites**						
Axial Volcano*^g^*	351	3.5–4.4	0.06–0.43	–	F	F
Endeavor Segment*^h^*	352	3.7–4.5	0.03–0.17	–	M, F	M, F
9°50′N EPR*^i^*	386	3.1–5.2	0.33–8.9	–	M	M
Kilo Moana*^j^*	304	3.9–4.1	0.22–0.50	–	M	M
Lucky Strike*^k^*	324	3.6–3.9	0.03–0.07	–	ND	M
Piccard*^l^*	398	3.1–3.3	18.9–20.7	<1–4.8	F	F
Guaymas Basin*^m^*	315	5.9	–	–	M	M
Loki’s Castle*^n^*	315	5.5–5.9	4.6–5.5	–	M	M
**Subduction-influenced (andesite/dacite-hosted) sites**						
Brothers Volcano*^o^*	303	2.1–4.4	0.01–0.02	–	M	M
Mariner Field*^p^*	359	2.4–2.7	0.03–0.18	–	ND	M
TOTO Caldera*^q^*	170	5.3	0.01	–	ND	M

The pH and concentrations of H_2_ and formate are for end-member (zero-Mg^2+^) hydrothermal fluid while the microbial data represent presence at the site in low-temperature fluids (F) and mineral samples (M). ND, not detected; –, not analyzed.

^a^Sometimes reported as mmol/kg or μmol/kg, respectively. ^b^Takai et al. (2004b), Gallant and Von Damm (2006), Kumagai et al. (2008), and Han et al. (2018); ^c^Perner et al. (2007); ^d^Schrenk et al. (2004), Brazelton et al. (2006), Lang et al. (2010), and Lang et al. (2012); ^e^Flores et al. (2011); ^f^McDermott et al. (2015) and Reveillaud et al. (2016); ^g^Ver Eecke et al. (2012), Topçuoğlu et al. (2016), and Fortunato et al. (2018); ^h^Ding et al. (2005), Ver Eecke et al. (2012), Anderson et al. (2013), and Lin et al. (2016); ^i^Von Damm and Lilley (2004), Ding et al. (2005), Kormas et al. (2006), McCliment et al. (2006), and Hou et al. (2020); ^j^Flores et al. (2012); ^k^Flores et al. (2011); ^l^Reveillaud et al. (2016) and McDermott et al. (2018); ^m^Von Damm et al. (1985) and Pagé et al. (2008); ^n^Jaeschke et al. (2012) and Baumberger et al. (2016); ^o^Takai et al. (2009) and Reysenbach et al. (2020); ^p^Takai et al. (2008) and Flores et al. (2012); ^q^Gamo et al. (2004) and Nakagawa et al. (2006).

The host rock in mafic and ultramafic sites have high concentrations of MgO and FeO, but they differ in their silica content, with ultramafic rocks having silica concentrations less than 45% (by weight), while mafic rocks have concentrations above 45%. Most abiotic formation of H_2_ in hydrothermal vents occurs by hydrothermal alteration of the ultramafic rock peridotite (i.e., serpentinization) (Table 1). Serpentinization occurs in environments with limited magma supply where peridotite is present in the rock hosting hydrothermal circulation and is mostly associated with ultramafic sites. Olivine and orthopyroxene, the most abundant minerals in peridotite, are unstable under hydrothermal conditions, which causes dissolution-reprecipitation reactions and the formation of serpentine, magnetite, and H_2_ [e.g., 6 (Mg, Fe)_2_SiO_4_ + 7 H_2_O → 3(Mg, Fe)Si_2_O_5_(OH)_4_ + Fe_3_O_4_ + H_2_] (Klein et al., 2020). *Methanococci* and *Thermococci* are common in most ultramafic-influenced hydrothermal sites except at the Lost City hydrothermal vent field (Table 1). At Lost City, the high pH hydrothermal fluids formed by low temperature serpentinization lead to calcium carbonate precipitation and very low dissolved inorganic carbon concentrations. This likely hinders the growth of autotrophs such as *Methanococci* and *Methanopyri* unless they can grow on an aqueous carbon source such as formate.

Serpentinization is inhibited by silica and is thus less common in mafic and felsic rocks (felsic rocks are > 65% silica by weight). In mafic (basalt)-hosted hydrothermal systems, the oxidation of ferrous iron minerals, such as pyrrhotite to pyrite (FeS + H_2_S → FeS_2_ + H_2_) and magnetite to hematite (2 Fe_3_O_4_ + H_2_O → 3 Fe_2_O_3_ + H_2_), and weathering of the ocean crust by oxygen-depleted water in the root zone of a hydrothermal system are also significant sources of H_2_ in hydrothermal systems (Klein et al., 2020). H_2_ and H_2_S concentrations are controlled by chemical equilibrium between fluid and the pyrite-pyrrhotite-magnetite mineral assemblages present. Most H_2_ and H_2_S fluid compositions fall close to the metastable extension of pyrite-pyrrhotite equilibrium (Klein et al., 2020). H_2_ concentrations in mafic hydrothermal fluids also increase significantly following a volcanic eruption as circulating fluids interact with newly injected rock (Lilley et al., 2003; Seewald et al., 2003; Von Damm and Lilley, 2004). Mafic hydrothermal vent sites generally tend to have *Thermococci* and *Methanococci* present (Table 1), especially following volcanic eruptions (Holden et al., 1998; Huber et al., 2002; Meyer et al., 2013), but *Methanococci* can become rare or undetectable during quiescent periods between eruptions when H_2_ concentrations decrease or in low H_2_ hydrothermal vents (Ver Eecke et al., 2009, 2012).

In contrast, hydrothermal vents that form along volcanic arcs at convergent plate boundaries have host rock with hydrous minerals, silica accumulation in aging oceanic crust, and more felsic character, such as dacite and andesite. The hydrothermal fluids from these rocks tend to have lower pH and lower H_2_ (Table 1). While *Thermococci* are generally present at these sites, *Methanococci* tend to be rare or undetectable (Table 1) likely due to the very low H_2_ concentrations (Ver Eecke et al., 2012).

Other more minor abiotic H_2_ contributions in hydrothermal vents come from magmatic degassing at low hydrostatic pressures (e.g., shallow vent sites) and radiolysis of water (Klein et al., 2020). Biotic sources of H_2_ at extremely thermophilic temperatures by *Thermococci* are described in Section “4. H_2_ production by *Thermococci*.”

## 3. H_2_ use by methanogens

Hydrogen is used by extremely thermophilic *Methanococci*, specifically, the genera *Methanocaldococcus* (T_opt_ 80–85°C), *Methanotorris* (T_opt_ 75–88°C), *Methanofervidicoccus* (T_opt_ 70°C), and *Methanothermococcus* (T_opt_ 65°C), and in the *Methanopyri*, which consists solely of *Methanopyrus kandleri* (T_opt_ 98°C) (Table 2).

**TABLE 2:** Growth characteristics of the classes *Methanococci* and *Methanopyri* and presence of genes for formate transport (FT), formate dehydrogenase (*fdh*), hydrogenases (*eha*, *ehb*, *frh*, *vhu*, *hmd*), and purine biosynthesis (*purT*, *purP*).

Organism	T_opt_ (°C)	Growth*	FT	*fdh*	*eha*	*ehb*	*frh*	*vhu*	*hmd*	*purT*	*purP*
*Methanocaldococcus jannaschii* JAL-1*^a^*	85	–		⚫	⚫	⚫	⚫⚫	⚫	⚫⚫⚫	⚫	⚫
*Methanocaldococcus bathoardescens* JH146*^b^*	82	–		⚫	⚫	⚫	⚫⚫	⚫	⚫⚫⚫	⚫	⚫
*Methanocaldococcus fervens* AG86*^c^*	85	ND	⚫	⚫	⚫	⚫	⚫	⚫	⚫⚫	⚫	⚫
*Methanocaldococcus infernus* ME*^d^*	85	–			⚫	⚫	⚫	⚫	⚫	⚫	⚫
*Methanocaldococcus vulcanius* M7*^e^*	80	–		⚫	⚫	⚫	⚫⚫	⚫⚫	⚫⚫⚫	⚫	⚫
*Methanotorris igneus* Kol 5*^f^*	88	–		⚫	⚫	⚫	⚫⚫⚫	⚫⚫	⚫⚫	⚫	⚫
*Methanotorris formicicus* Mc-S-70*^g^*	75	+	+	+	+	+	+	+	+	+	+
*Methanothermococcus okinawensis* IH1*^h^*	65	+	⚫	⚫	⚫	⚫	⚫⚫	⚫⚫	⚫	⚫	⚫
*Methanothermococcus thermolithotrophicus* SN 1*^i^*	65	+	+	+	+	+	+	+	+	+	+
*Methanofervidicoccus abyssi* HHB*^j^*	70	–			+	+	+	+	+	+	+
*Methanopyrus kandleri* AV19*^k^*	98	–		⚫	⚫		⚫⚫	⚫	⚫		⚫

The number of circles per column represents the number of times the gene(s) for that complex appears in the organism’s genome.

*Growth on formate; ND, not determined.

+ In protein columns indicates genes present in draft genome sequence.

References and genome accession numbers: ^a^Jones et al. (1983a), L77117; ^b^Ver Eecke et al. (2013), CP009149; ^c^Zhao et al. (1988), CP001696; ^d^Jeanthon et al. (1998), CP002009; ^e^Jeanthon et al. (1999), CP001787; ^f^Burggraf et al. (1990), CP002737; ^g^Takai et al. (2004a), AGJL01000032; ^h^Takai et al. (2002), CP002792; ^i^Huber et al. (1982), AQXV01000039; ^j^Sakai et al. (2019), BFAX0000000; ^k^Kurr et al. (1991), AE009439.

### 3.1. Hydrogenases in *Methanococci* and *Methanopyri*

**FIGURE 2:**
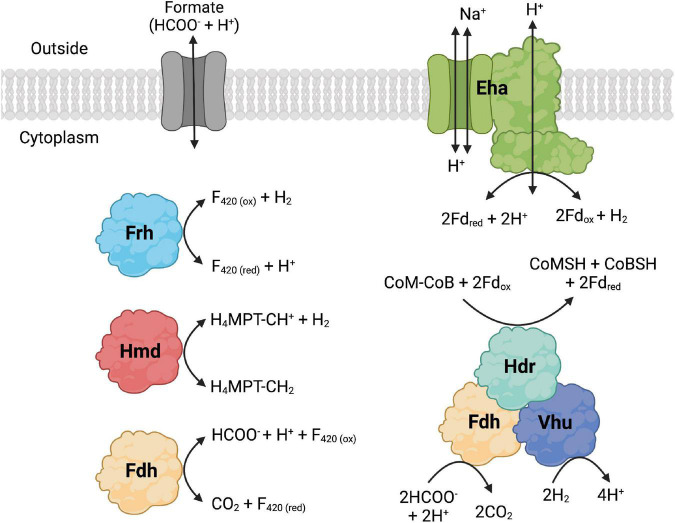
Formate dehydrogenase (Fdh), hydrogenase, and formate transporter proteins and their reactions that are found in *Methanococci* and *Methanopyri*. Fdh catalyzes the following formate oxidation reactions: cytoplasmic reduction of coenzyme F_420_ (F_420_) and cytoplasmic reduction of coenzyme M (CoM), coenzyme B (CoB), and ferredoxin (Fd). The hydrogenases catalyze the following H_2_ oxidation reactions: membrane-bound reduction of Fd (Eha), cytoplasmic reduction of F_420_ (Frh), cytoplasmic reduction of methenyl-tetrahydromethanopterin (CH-H_4_MPT) (Hmd), and cytoplasmic reduction of CoM, CoB, and Fd. F_420_, Fd, CoM, and CoB are cytoplasmic electron carriers. Methenyl-H_4_MPT is an intermediate of the Wood-Ljungdahl CO_2_ fixation pathway. Created with BioRender.com.

The whole genome sequences of 10 extremely thermophilic *Methanococci* plus *M. kandleri* were analyzed for known hydrogenases (see Supplementary materials). All 11 of the *Methanococci* and *Methanopyri* in the genome survey have at least one of the following hydrogenase genes (see Greening et al., 2016 for a review): (1) *eha* and *ehb* operons, which encode for membrane-bound multimeric hydrogenases that couple H_2_ oxidation to ferredoxin reduction and are H^+^/Na^+^ driven for anaplerotic (Eha) and anabolic (Ehb) purposes (Porat et al., 2006; Lie et al., 2012); (2) an *frh* operon, which encodes for a soluble complex that directly couples H_2_ oxidation to coenzyme F_420_ reduction (Hendrickson and Leigh, 2008); (3) an *hmd* gene, which encodes a soluble methylenetetrahydromethanopterin dehydrogenase that couples oxidation of H_2_ to the reduction of methenyltetrahydromethanopterin in the archaeal Wood-Ljungdahl CO_2_ fixation pathway (Hendrickson and Leigh, 2008); and (4) a *vhu* operon, which encodes for soluble heterodisulfide reductase-linked complexes that bifurcate electrons from H_2_ to heterodisulfide (coenzyme M-coenzyme B) and ferredoxin (Kaster et al., 2011). These hydrogenases are described and listed in Figure 2, Table 2, and Supplementary Table 1. Coenzyme F_420_, ferredoxin, coenzyme M, and coenzyme B are soluble electron carriers in methanogens (Thauer et al., 2008). Extremely thermophilic *Methanococci* and *Methanopyri* will often have two or three copies of the genes encoding these enzymes (Table 2 and Supplementary Table 1).

### 3.2. Growth of *Methanococci* on H_2_

The growth of natural assemblages of extremely thermophilic *Methanococci* in hydrothermal vent fluids from Axial Seamount is largely dependent on H_2_ availability and temperature (Topçuoğlu et al., 2016). The Monod kinetic half-saturation value (*K*_s_) for growth of extremely thermophilic methanogens was 27–66 μM with maximum methane production rates of 24–43 fmol CH_4_ produced cell^–1^ h^–1^ (Ver Eecke et al., 2012; Stewart et al., 2019). *Methanocaldococcus jannaschii* and *Methanothermococcus thermolithotrophicum* were shown to grow by interspecies H_2_ transfer when grown in co-culture with *Thermococcus celer*, *Thermococcus stetteri*, and *Pyrococcus furiosus* (Bonch-Osmolovskaya and Stetter, 1991). When *M. jannaschii* was grown in monoculture at high (80–83 μM) and low (15–27 μM) H_2_ concentrations and in co-culture with the hyperthermophilic H_2_ producer *Thermococcus paralvinellae* (representing very low H_2_ flux), growth and cell-specific CH_4_ production rates decreased with decreasing H_2_ availability (Topçuoğlu et al., 2019). However, the number of cells produced per mole of CH_4_ produced (i.e., cell yield) increased six-fold with decreasing H_2_ indicating increased growth efficiency when growth was limited by H_2_ (Topçuoğlu et al., 2019). Relative to high H_2_ concentrations, isotopic fractionation of CO_2_ to CH_4_ was 16‰ larger for cultures grown at low H_2_ concentrations and 45–56‰ larger in co-culture suggesting reversal of the Wood-Ljungdahl pathway during methanogenesis with low H_2_ flux (Valentine et al., 2004; Topçuoğlu et al., 2019). While all four types of hydrogenases were synthesized by *M. jannaschii* with high and low H_2_ flux, transcript levels of *hmd* and *eha* decreased with decreasing H_2_ availability (Topçuoğlu et al., 2019).

## 4. H_2_ production by *Thermococci*

Hydrogen is produced by *Thermococci*, specifically, the genera *Thermococcus* (T_opt_ 75–90°C), *Palaeococcus* (T_opt_ 83°C), and *Pyrococcus* (T_opt_ 96–105°C) (Table 3).

**TABLE 3:** Growth characteristics of the class *Thermococci* and presence of genes for formate transport (FT), formate dehydrogenase operons (*fhl, nfd*), and individuals (*fdhA*) with neighboring hydrogenase operons, individual hydrogenase operons (*mbh*, *sh*, *frh*, *codh*), and purine biosynthesis (*purT*, *purP*).

Organism	T_opt_ (°C)	H_2_ ↔ formate*	FT	Group 1A: *frh-fhl-mbh*	Group 1B: *frh-nfd-mbh*	Group 2: *nfd-sh*	Group 3: *fhl-sh*	Group 4: *fhl* only	Group 5: *fdhA* only	*Mbh*	*sh*	*frh*	*codh*	*purT*	*purP*
*Thermococcus paralvinellae* ES1^d^	82	+*^b,c^*	⚫	⚫				⚫		⚫⚫	⚫	⚫	⚫		
*Thermococcus barophilus* CH5^a^	80	+*^a,c^*	⚫	⚫			⚫			⚫⚫	⚫◯	⚫	⚫		⚫
*Thermococcus onnurineus* NA1^e^	80	+*^a,c^*	⚫⚫	⚫		⚫		⚫		⚫	⚫⚫	⚫	⚫		
*Thermococcus gammatolerans* EJ3^f^	88	+*^a,c^*	⚫	⚫				◯	⚫	⚫		⚫	◯	⚫	⚫
*Thermococcus piezophilus* CDGS^g^	75	+*^c^*	⚫	⚫		⚫		⚫		⚫	⚫⚫	⚫		⚫	⚫
*Thermococcus cleftensis* CL1^h^	88	+*^c^*	⚫		⚫				⚫	⚫	⚫⚫	⚫		⚫	⚫
*Thermococcus nautili* 30-1^i^	88	+*^c^*	⚫		⚫				⚫	⚫	⚫⚫	⚫		⚫	⚫
*Thermococcus kodakarensis* KOD1^j^	85	+*^c^*	⚫		Δ				⚫	⚫	⚫			⚫	⚫
*Thermococcus chitonophagus* GC74^k^	85	−*^a,c^*						⚫	⚫	⚫	⚫⚫			⚫	⚫
*Thermococcus eurythermalis* A501^l^	85	−*^c^*					⚫	⚫	⚫	⚫	⚫	⚫		⚫	⚫
*Thermococcus pacificus* P-4^m^	88	−*^c^*					⚫			⚫	⚫	⚫		⚫	⚫
*Thermococcus litoralis* NC-S^n^	88	−*^c^*					⚫		⚫	⚫⚫	⚫⚫			⚫	⚫
*Thermococcus barophilus* MP^o^	85	−*^c^*							⚫	⚫⚫	⚫⚫		⚫	⚫	⚫
*Thermococcus sibiricus* MM 739^p^	78	−*^a,c^*							⚫	⚫⚫	⚫⚫			⚫	⚫
*Thermococcus guaymasensis* TYS^q^	88	−*^c^*						◯		⚫	⚫⚫	⚫	⚫	⚫	⚫
*Thermococcus celer* Vu 13^r^	88	−*^a,c^*							⚫	⚫	⚫⚫			⚫	⚫
*Thermococcus peptonophilus* OG-1^s^	90	−*^a,c^*						◯	⚫	⚫	⚫⚫			⚫	⚫
*Thermococcus barossii* SHCK-94^t^	83	−*^c^*							⚫	⚫⚫	⚫⚫		◯	⚫	⚫
*Thermococcus siculi* RG-20^u^	85	−*^c^*							⚫	⚫⚫	⚫⚫		◯	⚫	⚫
*Thermococcus radiotolerans* EJ2^v^	88	−*^c^*							⚫	⚫⚫	⚫⚫		◯	⚫	⚫
*Thermococcus profundus* DT 5432^w^	80	−*^a,c^*							⚫	⚫	⚫⚫		◯	⚫	⚫
*Thermococcus indicus* IOH1^x^	80	ND							⚫	⚫⚫	⚫⚫	◯		⚫	⚫
*Thermococcus camini* IRI35c^y^	80	–							⚫	⚫⚫	⚫⚫			⚫	⚫
*Thermococcus gorgonarius* W-12^z^	88	−*^c^*						◯		⚫	⚫				
*Palaeococcus pacificus* DY20341^aa^	83	+*^c^*	⚫			⚫				⚫⚫	⚫⚫			⚫	⚫
*Pyrococcus kukulkanii* NCB100^ab^	105	+*^c^*	⚫	⚫				⚫	⚫	⚫	⚫⚫	⚫		⚫	⚫
*Pyrococcus yayanosii* CH1^ac^	98	+*^c^*	⚫	⚫						⚫	⚫⚫	⚫		⚫	⚫
*Pyrococcus abyssi* GE5^ad^	96	−*^c^*						⚫	⚫	⚫	⚫⚫			⚫	⚫
*Pyrococcus furiosus* Vc1^ae^	100	−*^c^*							⚫	⚫	⚫⚫			⚫	⚫
*Pyrococcus horikoshii* OT3^af^	98	−*^c^*							⚫	⚫	⚫			⚫	⚫

The number of circles per column represents the number of times the gene(s) for that protein or operon appears in the organism’s genome. Open circles represent incomplete operons; the open triangle, and *sh-nfd-mbh* operon configuration.

*Conversion of formate to H_2_ or H_2_ to formate; ^a^Kim et al. (2010); ^b^Topçuoğlu et al. (2018); ^c^Le Guellec et al. (2021); ND, not determined. References and genome accession numbers: ^d^Pledger and Baross (1989), CP006965; ^a^Kim et al. (2010), CP013050; ^e^Bae et al. (2006), CP000855; ^f^Jolivet et al. (2003), CP001398; ^g^Dalmasso et al. (2016), CP015520; ^h^Holden et al. (2001), CP003651; ^i^Gorlas et al. (2014), CP007264; ^j^Atomi et al. (2004), AP006878; ^k^Huber et al. (1995), LN999010; ^l^Zhao et al. (2015), CP008887; ^m^Miroshnichenko et al. (1998), CP015102; ^n^Belkin et al. (1985), CP006670; ^o^Marteinsson et al. (1999), CP002372; ^p^Miroshnichenko et al. (2001), CP001463; ^q^Canganella et al. (1998), CP007140; ^r^Zillig et al. (1983), CP014854; ^s^González et al. (1995), CP014750; ^t^Duffaud et al. (1998), CP015101; ^u^Grote et al. (1999), CP015103; ^v^Jolivet et al. (2004), CP015106; ^w^Kobayashi et al. (1994), CP014862; ^x^Lim et al. (2020), CP040846; ^y^Courtine et al. (2021), LR881183; ^z^Miroshnichenko et al. (1998), CP014855; ^aa^Zeng et al. (2013), CP006019; ^ab^Callac et al. (2016), CP010835; ^ac^Birrien et al. (2011), CP002779; ^ad^Erauso et al. (1993), AL096836; ^ae^Fiala and Stetter (1986), AE009950; ^af^González et al. (1998), BA000001.

### 4.1. Hydrogenases in *Thermococci*

**FIGURE 3:**
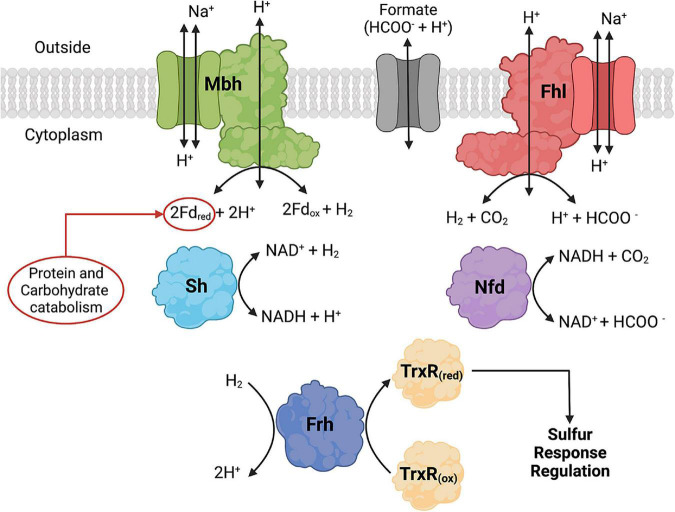
Formate dehydrogenase, hydrogenase, and formate transporter proteins and their reactions that are found in *Thermococci*. Formate hydrogenlyase (Fhl) catalyzes membrane-bound oxidation of formate to H_2_ and CO_2_. NAD(P)H: formate dehydrogenase (Nfd) catalyzes cytoplasmic oxidation of formate coupled with reduction of NAD(P)^+^. The hydrogenases catalyze the following reactions: membrane-bound oxidation of ferredoxin (Fd) coupled with H_2_ production (Mbh), cytoplasmic H_2_ oxidation coupled with reduction of NAD(P)^+^ (Sh), and cytoplasmic H_2_ oxidation (Frd) coupled with reduction of thioredoxin reductase (TrxR). Fd and NAD(P)H are cytoplasmic electron carriers. TrxR is part of the redox cascade for sulfur response regulation using SurR. Created with BioRender.com.

The whole genome sequences of 30 *Thermococci* were analyzed for known hydrogenases (see Supplementary materials). All 30 *Thermococci* analyzed have at least one of the following hydrogenase operons: (1) An *mbh* operon, which encodes for a membrane-bound hydrogenase that couples oxidation of ferredoxin to H_2_ evolution with concomitant H^+^/Na^+^ translocation across the membrane using antiporters (Sapra et al., 2003); (2) an *sh* operon, which encodes for a soluble sulfhydrogenase that couples oxidation of H_2_ oxidation to the reduction of NAD(P)^+^ (Van Haaster et al., 2008); (3) an *frh* operon, which encodes for cytoplasmic coenzyme F_420_ reducing-type hydrogenase that oxidizes H_2_ and passes electrons to a thioredoxin reductase (Jung et al., 2020); and (4) a *codh* operon, which encodes for a membrane-bound hydrogenase that couples oxidation of CO to H_2_ evolution with concomitant H^+^/Na^+^ translocation across the membrane using antiporters (Bae et al., 2012; Moon et al., 2012). These hydrogenases are described and listed in Figure 3, Table 3, and Supplementary Table 2.

All *Thermococci* have at least one *mbh* operon and all but one have at least one *sh* operon (Table 3). These enzymes are the core hydrogenases for *Thermococci* (Schut et al., 2012; Boyd et al., 2014). Twelve of the 30 *Thermococci* in the survey have *frh* operons. Five of the 30 *Thermococci* have *codh* operons. It was shown that the growth of *Thermococcus* sp. strain AM4 and *Thermococcus onnurineus* can be supported by CO with concomitant H_2_ production (Sokolova et al., 2004; Bae et al., 2012; Moon et al., 2012), although the physiological role of this enzyme in *Thermococcus* is yet to be determined for growth in its natural environment.

### 4.2. Growth of *Thermococci* with and without S^0^

In *Thermococci*, the reduction of S^0^ is the preferred route for electron disposal over the reduction of H^+^ to H_2_. In *P. furiosus*, the presence of S^0^ in growth media resulted in decreases in Mbh and Sh hydrogenase specific activities, each by an order of magnitude (Adams et al., 2001). There was an immediate downregulation of *mbh* and an upregulation of *mbs* (membrane-bound sulfane reductase) (Wu et al., 2018) and *nsr* (NAD(P)H:S^0^ reductase) in *P. furiosus* when S^0^ was added to growth medium (Schut et al., 2001, 2007). A sulfur response regulator protein (SurR) was identified as the transcription factor regulating hydrogenase and sulfur responsive genes (Lipscomb et al., 2009, 2017). The proposed model suggests that SurR contains a redox-active cysteine disulfide that can reduce S^0^ to H_2_S (Yang et al., 2010). SurR is reduced in a redox cascade involving NAD(P)H-dependent thioredoxin reductase (TrxR) and protein disulfide oxidoreductase (Pdo) as the electron donors (Lim et al., 2017). In the absence of S^0^, SurR remains reduced, binds to GTTn_3_AAC(n_5_GTT), promotes the transcription of *mbh* and *sh* genes, and represses the expression of *mbs* and *nsr* genes (Lipscomb et al., 2009). *Thermococci* with the Frh hydrogenase also can reduce TrxR using H_2_ as the electron donor (Jung et al., 2020).

## 5. Abiotic formate in hydrothermal vents

### 5.1. Formate production in hydrothermal fluids

Abiotic formation of formate, carbon monoxide, methane, and hydrocarbons in hydrothermal vents is of interest as potential growth substrates for microbes. Methane and hydrocarbons in vents were suggested to form through Fischer-Tropsch type reactions [(2*n* + 1)H_2_ + *n*CO → C*_*n*_*H_2_*_*n*_*_+2_ + *n*H_2_O] or leach from fluid inclusions in plutonic rocks (Berndt et al., 1996; Horita and Berndt, 1999; McCollom and Seewald, 2001; McDermott et al., 2015). In contrast to hydrocarbons, there is a strong thermodynamic drive toward rapid C-H-O equilibrium in hydrothermal fluids within hours to days. Kinetic barriers preclude the formation of CH_4_ in this equilibrium (Shock, 1990). This permits the creation of metastable formate species (H_2_ + CO_2_ ↔ HCOOH), CO (HCOOH ↔ CO + H_2_O), formaldehyde (HCOOH + H_2_ ↔ CH_2_O + H_2_O), and methanol (CH_2_O + H_2_ ↔ CH_3_OH) through the sequential reduction of CO_2_ using H_2_ as the reductant (Seewald et al., 2006).

The abundance of formate in chemical equilibrium with dissolved inorganic carbon is strongly dependent on H_2_ concentration, pH, and temperature (McCollom and Seewald, 2003; Seewald et al., 2006). In a gold-titanium reaction cell, HCOO^–^ was formed from CO_2_ at 300°C and 350 bar in less than 24 h from H_2_ generated from hydrothermal alteration of olivine serving as the reductant (McCollom and Seewald, 2001). In a separate study, incubation of a 175 mmol/kg HCOOH solution at 300°C and 350 bar in the gold reaction cell led to near complete conversion to H_2_ and CO_2_ within 48 h, CO reached 0.83 mmol/kg, and HCOO^–^ + HCOOH (or ΣHCOOH) decreased to 0.38 mmol/kg (Seewald et al., 2006). Reducing the temperature to 200°C and then to 150°C each led to an increase in ΣHCOOH, a decrease in CO, and C-H-O equilibrium within 115 h and 71 h, respectively. Injection of 172 mmol/kg CO led to production of H_2_, ΣCO_2_, and ΣHCOOH, and decreasing CO. Alkaline conditions favored the formation of HCOOH, HCO_3_^–^, and CO_3_^2–^ (Seewald et al., 2006). Therefore, the abundance of formate, CO, and CH_3_OH in seafloor hydrothermal systems will be regulated by the residence times of fluids in reactions zones, and physical and chemical conditions in the subsurface environments.

Formate is also generated across a pH gradient of more than three pH units using a mineral precipitate bridge at the interface of two fluids (Hudson et al., 2020). This may be relevant to the formation of formate on the early Earth or possibly in extraterrestrial oceans where high pH serpentinized fluids are emitted into an acid ocean. Under standard conditions, the generation of formate from H_2_ and CO_2_ is not thermodynamically favorable. However, H_2_ in synthetic alkaline vent fluid (pH 12.3) passed electrons to dissolved CO_2_ in a synthetic acid ocean (pH 3.9) at 25°C through a Fe(Ni)S mineral interface generating 1.5 μM HCOO^–^ in the ocean fluid (Hudson et al., 2020). Isotopic labeling showed that protonation occurred using H_2_O on the ocean side of the interface, not H_2_ on the vent side. Weakening the pH gradient led to decreased concentrations of HCOO^–^ produced. Nickel in the precipitate is a crucial part of the reduction mechanism as HCOO^–^ yield dropped below detection without Ni in the ocean precipitation fluid.

### 5.2. Formate concentrations in hydrothermal fluids

There have been very few measurements of formate in natural hydrothermal fluids due in part to the analytical difficulty of measuring formate at low concentrations (Schink et al., 2017). Formate has been measured mostly at sites with high H_2_ concentrations such as at the Lost City, Von Damm, and Piccard hydrothermal vent sites and were 36–669 μM (Table 1). Formate and H_2_ were also measured at Snake Pit and TAG hydrothermal vents, which are mafic hydrothermal vents on the Mid-Atlantic Ridge, where formate concentrations were 1–2 nM and H_2_ concentrations were 0.08–2.4 μM (Konn et al., 2022). At ultramafic sites, formate concentrations are generally 10–100 fold lower than that of H_2_ at the same site (Lang et al., 2010; McDermott et al., 2015) while at mafic sites the formate concentration is often more than 1,000 fold lower than the H_2_ concentration (McDermott et al., 2018; Konn et al., 2022).

## 6. Formate use by methanogens

### 6.1. Free formate use for *de novo* purine biosynthesis

**FIGURE 4:**
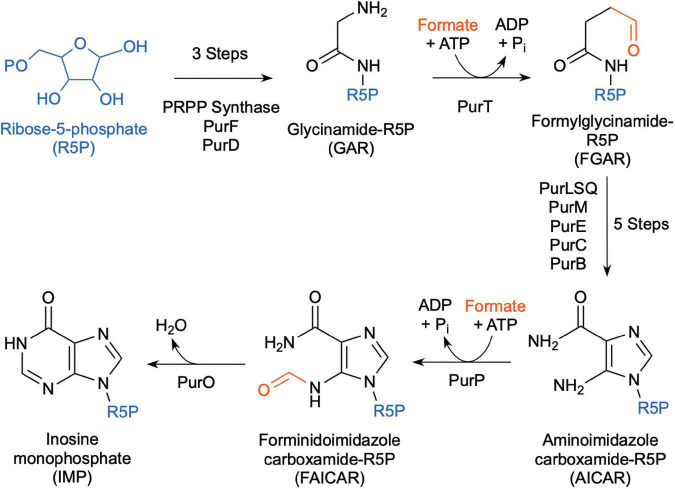
Biochemical pathway for *de novo* purine biosynthesis using free formate as the source of the formyl group (after Brown et al., 2011).

*Methanocaldococcus jannaschii* was shown to incorporate ^14^C-formate into biomass during growth (Sprott et al., 1993), which may be used in part for *de novo* purine biosynthesis. Inosine monophosphate (IMP) is a precursor for adenine and guanine synthesis for purine biosynthesis and is made from ribose-5-phosphate (Figure 4). In most organisms, the pathway intermediates glycinamide-ribose-5-phosphate (GAR) and aminoimidazole carboxamide-ribose-5-phosphate (AICAR) are formylated using N^10^-formyl-tetrahydrofolate as the formyl donor. However, the genes for these enzymes are absent in *Methanococci* and *Methanopyri* and are replaced with genes that encode for enzymes that use free formate and energy from ATP to formylate their substrates (White, 1997; Brown et al., 2011). These enzymes are formylglycinamide-ribose-5-phosphate synthetase (PurT) and forminido imidazole carboxamide-ribose-5-phosphate synthetase (PurP) (Figure 4). *M. jannaschii* was shown to have PurP activity and that it produced free ^13^C-formate in the cell when incubated with H_2_ and H^13^CO_3_ (Ownby et al., 2005). Herein, a genome survey of the eleven extremely thermophilic methanogens showed that all the organisms have homologs for *purP* and all but *M. kandleri* have homologs for *purT* (Table 2 and Supplementary Table 1). This suggests that these organisms have a mechanism for formate synthesis.

### 6.2. Formate dehydrogenases in *Methanococci* and *Methanopyri*

Nine of the 11 *Methanococci* and *Methanopyri* genomes have genes that encode for a cytoplasmic formate dehydrogenase (Table 2 and Supplementary Table 1). Formate dehydrogenases catalyze the reversible oxidation of formate to CO_2_ using various electron acceptors. The catalytic α subunit (FdhA) contains tungsten, selenocysteine, and a (Fe_4_-S_4_) cluster as cofactors while the β subunit (FdhB) contains three (Fe_4_-S_4_) clusters (Niks and Hille, 2019). FdhAB in *Methanococci* and *Methanopyri* is homologous to two formate dehydrogenases in the mesophilic methanogen *Methanococcus maripaludis*, also a *Methanococci*, that use coenzyme F_420_ as their redox partner (Figure 2; Wood et al., 2003; Lupa et al., 2008). *M. maripaludis* grows hydrogenotrophically on H_2_ and CO_2_ but also grows on formate in their absence (Jones et al., 1983b). When *fdhA1* was mutated in *M. maripaludis*, the organism was unable to grow on formate and formate dehydrogenase activity in cell extracts was undetectable (Lupa et al., 2008). Observations with hydrogenase mutants in *M. maripaludis* suggest that coenzyme F_420_ is an intermediate in formate-to-H_2_ conversion (Lupa et al., 2008). An *M. maripaludis*Δ*fdhA1*Δ*fdhA2* double mutant grown in purine-free defined medium grew as well as the wild-type strain suggesting that formate dehydrogenase is not essential for *de novo* purine biosynthesis (Wood et al., 2003). The absence of *fdhAB* genes in *Methanocaldococcus infernus* and *Methanofervidicoccus abyssi* (Table 2 and Supplementary Table 1) also supports the idea that formate dehydrogenase is not essential for purine biosynthesis. However, it is likely that H_2_ and coenzyme F_420_ are electron donors for formate production and can help meet the cellular demand for formate for purine biosynthesis.

The formate dehydrogenase (FdhA1B1) from *M. maripaludis* also forms an enzyme complex with heterodisulfide reductase, the soluble hydrogenase Vhu, and formylmethanofuran dehydrogenase (Figure 2; Costa et al., 2010). It was necessary for the organism’s growth on formate but not on H_2_ (Costa et al., 2010). Therefore, in addition to coenzyme F_420_ reduction, this formate dehydrogenase also oxidizes formate to reduce the heterodisulfide coenzyme M-coenzyme B and ferredoxin through electron bifurcation. Coenzyme M, coenzyme B, and ferredoxin are cytoplasmic electron carriers in these methanogens. Expression of the second formate dehydrogenase gene (*fdhA2*) in *M. maripaludis* increased when cells were grown under H_2_ limited conditions but was unchanged under formate limited conditions (Costa et al., 2013) and was not required for growth on formate (Lupa et al., 2008) suggesting that this isoenzyme may have a separate physiological function.

### 6.3. Formate transporters in *Methanococci*

For extremely thermophilic methanogens, it appears that a formate transporter is required for growth on formate. Formate transporters import or export formate across the cytoplasmic membrane and require co-translocation of a H^+^ (Figure 2). Three thermophilic methanogens in our survey (*Methanotorris formicicus*, *Methanothermococcus okinawensis*, and *Methanothermococcus thermolithotrophicus*) grew on formate in the absence of H_2_ and CO_2_ but not any of the other methanogens examined (Table 2). Each of these methanogens that grew on formate has a gene that encodes for a membrane-bound formate transporter (*fdhC*) in its genome, which is absent in all other methanogens examined, except for *Methanocaldococcus fervens* which was not tested for growth on formate (Table 2 and Supplementary Table 1). *M. maripaludis* has an *fdhC* gene in an operon with *fdhA1B1* (Sattler et al., 2013). In *M. fervens* and *M. okinawensis*, the formate transporter gene *fdhC* appears to be in the same operon as *fdhAB* suggesting they are co-transcribed (Supplementary Table 1).

## 7. Formate use by *Thermococci*

### 7.1. Free formate use for *de novo* purine biosynthesis

Like *Methanococci*, all *Thermococci* lack the enzymes that use N^10^-formyl-tetrahydrofolate as the formyl donor for *de novo* purine biosynthesis (Brown et al., 2011). Instead, most *Thermococci* use formate-dependent enzymes (PurT and PurP) for *de novo* purine biosynthesis (Figure 4, Table 3, and Supplementary Table 2). Therefore, they depend on a source of free formate in the cell for *de novo* synthesis. However, some *Thermococcus* species (*T. paralvinellae*, *T. barophilus* CH5, *T. onnurineus*, and *T. gorgonarius*) lack most or all the genes for the purine biosynthesis pathway (Brown et al., 2011) and likely rely on environmental sources of purines.

### 7.2. Formate dehydrogenases in *Thermococci*

**FIGURE 5:**
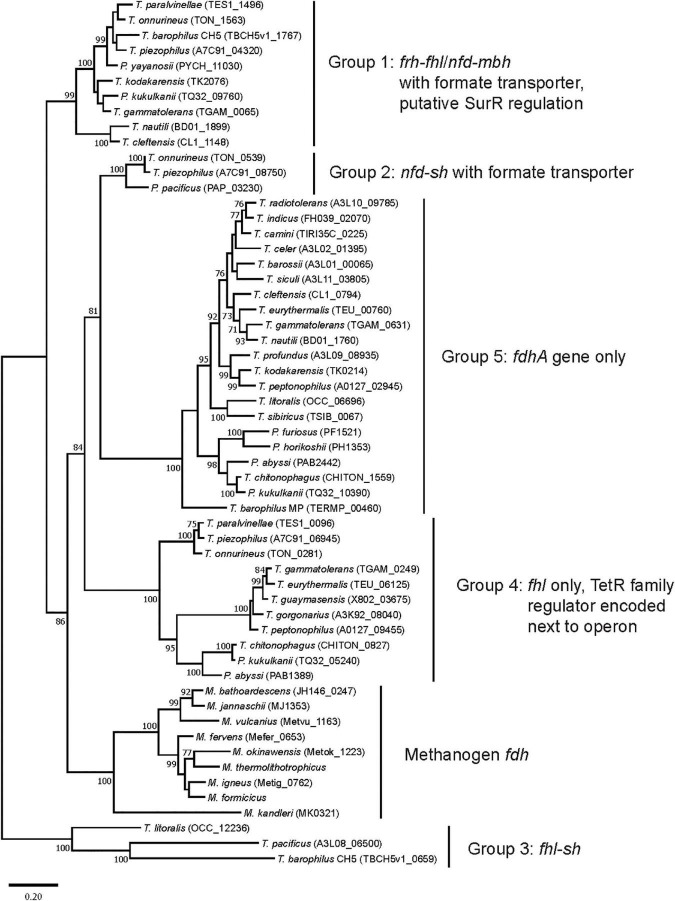
Phylogenetic tree based on catalytic subunit alpha (FdhA) for the various formate dehydrogenases found in extremely thermophilic *Methanococci*, *Methanopyri*, and *Thermococci*. The phylogeny of FdhA was inferred by using a maximum likelihood method and Jones-Taylor-Thornton (JTT) matrix-based modeling (Jones et al., 1992). After 1000 bootstrap constructions, the tree with the highest log likelihood (–31,270.37) is shown, with values next to nodes indicating the percentage of reconstructions in which the topology was preserved (values < 70% are omitted for clarity). There were a total of 736 positions in the final dataset. Branch lengths are to scale and indicate the number of substitutions per site. GenBank/EMBL/DDBJ open reading frame numbers are included in parentheses. Evolutionary analyses were conducted in MEGA11 (Tamura et al., 2021). Clade associations with operon arrangements on the genomes and the presence of a formate transporter or putative regulatory elements are shown.

**FIGURE 6:**
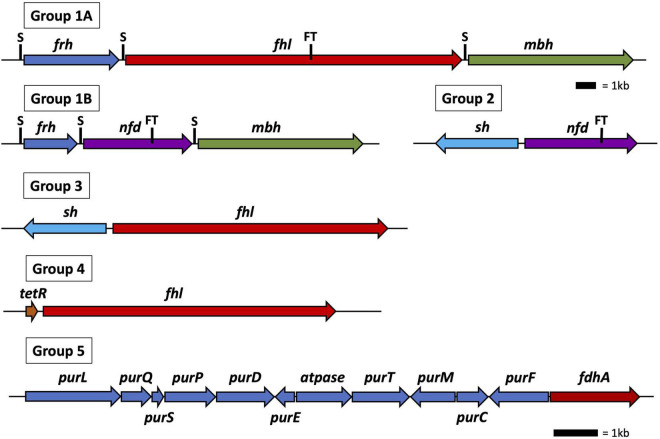
Operon and gene maps for *Thermococci* containing operons for formate hydrogenlyase (*fhl*), NAD(P)H: formate dehydrogenase (*nfd*), membrane hydrogenase (*mbh*), soluble hydrogenase (*sh*), and F_420_-reducing-like hydrogenase (*frh*) (Groups 1–4) and genes for the catalytic subunit of formate dehydrogenase (*fdhA*), and purine biosynthesis (*pur*) (Group 5). Also shown are the locations of SurR binding sites (S), the *tetR* gene for transcriptional regulation, and the operons containing a formate transporter (FT) gene. The top scale bar is for the Group 1–4 operons; the bottom scale bar, the genes for Group 5.

All 30 *Thermococci* genomes have at least one copy of the gene that encodes for the catalytic α subunit of formate dehydrogenase (FdhA) either in the form of formate hydrogenlyase, NAD(P)^+^-dependent formate dehydrogenase, or the catalytic subunit alone (Table 3 and Supplementary Table 2). The phylogeny of FdhA in extremely thermophilic *Methanococci*, *Methanopyri*, and *Thermococci* showed one clade for *Methanococci* and *Methanopyri* and five clades among the *Thermococci* (Figure 5). In *Thermococci*, hydrogenase operons often flank *fdhA*-containing operons in the genome (Figure 6 and Supplementary Table 2) suggesting a close association between formate and H_2_ in these organisms. In Groups 1 and 2 in Figure 5, *fdhA* was encoded in an operon with a formate transporter gene. For Group 1, in nearly all instances, the *fdhA*-containing operon was immediately downstream from an *frh* operon and immediately upstream from one or two *mbh* operons on the same DNA strand suggesting that they may be co-transcribed (Figure 6). In Group 1A, *fdhA* was encoded in a formate hydrogenlyase (*fhl*) operon (Kim et al., 2010; Topçuoğlu et al., 2018; Le Guellec et al., 2021; Table 3; Supplementary Table 2). This enzyme reversibly couples formate oxidation to H_2_ evolution on the cytoplasmic membrane with concomitant H^+^/Na^+^ translocation across the membrane *via* antiporter modules (Figure 4; Kim et al., 2010; Lim et al., 2014). In Group 1B, *fdhA* was encoded in a NAD(P)^+^-dependent formate dehydrogenase (*nfd*) operon (Figure 6). This soluble enzyme catalyzes the reversible oxidation of formate using NAD(P)^+^ or ferredoxin as its redox partner (Le Guellec et al., 2021; Yang et al., 2022; Figure 4). In Group 2, *fdhA* was encoded in an *nfd* operon but neighbored an *sh* operon in the genome instead of *frh* and *mbh* operons (Figure 6). These *nfd* and *sh* operons are transcribed in opposite directions from the same intergenic spacer region.

The *fdhA* from Groups 3 and 4 are in *fhl* operons that lack a formate transporter gene. In Group 3, the *fhl* operon was next to an *sh* operon (Figure 6). These *fhl* and *sh* operons are transcribed in opposite directions from the same intergenic spacer region. In Group 4, the *fhl* operon did not neighbor any hydrogenase operons in the genome, and in Group 5 the *fdhA* gene was the only formate dehydrogenase-related gene present in the genome (Figure 6). Often these solo genes in Group 5 are near the purine biosynthesis genes in genome sequences (Supplementary Table 2). In *T. sibiricus*, nearly all the genes for *de novo* purine biosynthesis (*purFCMTEDPSQL*) and *fdhA* are next to each other in the genome, although they are not all on the same DNA strand (Figure 6). In these organisms, it is unknown if *fdhA* alone encodes for a functional formate dehydrogenase or what the redox partner is for this putative enzyme. However, it is plausible that it might be used to produce formate for purine biosynthesis when other formate dehydrogenases and formate transport proteins are absent.

### 7.3. Formate transporters in *Thermococci*

Under defined growth conditions, 11 of the 30 *Thermococci* strains analyzed either oxidized added formate as an energy source (plus trace levels of organic compounds as a carbon source) and produced H_2_ (Kim et al., 2010; Topçuoğlu et al., 2018) or secreted formate when grown on organic compounds in the presence of high background H_2_ and the absence of added formate (Hensley et al., 2016; Topçuoğlu et al., 2018; Le Guellec et al., 2021). These 11 strains are the only *Thermococci* in the survey that have a formate transporter gene (Table 3). The other 19 *Thermococci* lack this gene and were unable to grow on formate or secrete formate (Kim et al., 2010; Le Guellec et al., 2021). Therefore, it appears that a formate transporter is required for *Thermococci* to secrete formate or, like *Methanococci*, for growth of *Thermococci* on formate. The presence of a formate transporter gene or transcript should be a criterion when determining if *Methanococci* or *Thermococci* are potentially using or producing formate in their natural habitat.

### 7.4. Formate production versus consumption by *Thermococci* in nature

The standard Gibbs energy for interconversion between formate and H_2_ + CO_2_ is small; therefore, the direction of the reaction is highly dependent upon the relative concentrations of formate and H_2_ in the environment (Schink et al., 2017; Le Guellec et al., 2021). Le Guellec et al. (2021) calculated that CO_2_ reduction to formate using H_2_ is thermodynamically more favorable than formate oxidation to H_2_ and CO_2_ at Lost City, Von Damm, Rainbow, Lucky Strike, Snake Pit, and Ashadze 1 hydrothermal vent sites based on relative formate and H_2_ concentrations in hydrothermal fluids. The physiological response of *Thermococcus* is in keeping with this idea. Growth of *T. paralvinellae* on a sugar or peptides when sparged with H_2_ led to higher levels of *fhl1* expression and higher formate secretion relative to cultures sparged with N_2_ (Topçuoğlu et al., 2018). It was concluded that *fhl* and *nfd* expression in *Thermococci* is primarily for the purpose of ameliorating H_2_ inhibition rather than for growth on formate (Topçuoğlu et al., 2019; Le Guellec et al., 2021). *Thermococci* would require an environment where formate concentrations exceed H_2_ concentrations to grow on formate. The formate produced by *Thermococci* may supplement the growth of *Methanococci* even when *Thermococci* produce H_2_, as is observed with fermenter-methanogen relationships in mesophilic environments (Schink et al., 2017).

## 8. Transcriptional regulation of formate dehydrogenase genes

### 8.1. Transcriptional regulation in *Methanococci*

Formate consumption in *Methanococci* is closely associated with H_2_ use in the cell. Therefore, a question that arises is whether formate or H_2_ regulates *fdhAB* expression in these organisms. The thermophilic methanogen *Methanobacterium thermoformicicum* grows on H_2_ and CO_2_ as well as separately on formate. It has a formate transporter gene (*fdhC*) directly upstream of its formate dehydrogenase genes (*fdhAB*) (Nolling and Reeve, 1997). Transcripts of *fdhCAB* were present in *M. thermoformicicum* at all growth stages when grown on formate. When grown on H_2_ and CO_2_, *fdhCAB* transcripts were barely detectable in early exponential growth phase but increased dramatically as cells approached late exponential growth phase in a closed batch system when H_2_ became more limiting. Similarly, *fdh* expression in *M. maripaludis* was controlled by the presence of H_2_ and not formate (Wood et al., 2003). Using *fdhC-lacZ* gene fusions, β-galactosidase activity increased in *M. maripaludis* cells grown on H_2_ and CO_2_ as they approached late exponential growth phase, again when H_2_ became limiting. When grown on formate, β-galactosidase activity was higher in cells with N_2_ and CO_2_ in the headspace relative to those with H_2_ and CO_2_ in the headspace. β-galactosidase activity increased in cells grown on formate plus H_2_ and CO_2_ after the H_2_ and CO_2_ was replaced mid-growth phase with N_2_ and CO_2_.

In *M. maripaludis*, genes for a putative response regulator and a histidine kinase are directly upstream of *fdhC*, which is three genes upstream of *fdhA1B1* and part of a putative five-gene operon (Sattler et al., 2013). Random mutagenesis showed that disruption of this putative response regulator led to slower growth of *M. maripaludis* on formate relative to the wild type. It also led to increased *fdhA1* transcriptional abundance regardless of whether H_2_ and CO_2_ or formate was the growth substrate. Impairment of derepression of the *fdhC-fdhA1B1* operon is a plausible explanation (Sattler et al., 2013). Therefore, H_2_ present at high concentrations may interact with the histidine kinase and activate the response regulator in a two-component regulatory system that represses *fdhC-fdhA1B1* expression, which is derepressed when H_2_ levels are low or absent.

### 8.2. Transcriptional regulation in *Thermococci*

Very little is known about transcriptional regulation of the *fhl* and *nfd* operons in *Thermococci*. Group 1 *Thermococci* genomes (Figure 6) encode syntenic *frh*, either *fhl* or *nfd*, and *mbh* operons with a formate transporter gene encoded in the *fhl* or *nfd* operon (Figure 6). These *frh*, *fhl*, *nfd*, and *mbh* operons each have GTTn_3_AAC(n_5_GTT) in their promoter region just upstream of BRE/TATA RNA polymerase binding sites suggesting they are also regulated and promoted by the sulfur response regulator protein SurR (see Section “4.2. Growth of *Thermococci* with and without S^0^”). Furthermore, Frh was shown to oxidize H_2_ and reduce TrxR (Jung et al., 2020), which reduces SurR *via* Pdo, suggesting that it might serve as a regulatory hydrogenase that promotes *frh*, *fhl*, *nfd*, and *mbh* expression when H_2_ concentrations increase in the cell. Therefore, like *Methanococci*, H_2_ abundance appears to regulate formate use in *Thermococci*. A remaining question is whether formate also regulates gene expression in *Thermococci*. In *T. paralvinellae*, expression of the Group 1A *fhl* operon containing the formate transporter gene increased when cells were grown on formate relative to growth on maltose or peptides while expression of *mbh* either remained unchanged or decreased (Topçuoğlu et al., 2018). This suggests that in addition to SurR regulation, formate either directly or indirectly regulates gene expression in *T. paralvinellae* as well. Validation and the mechanism of this putative regulation is yet to be determined.

None of the promoter regions for the *nfd*, *fhl*, or *sh* operons in Groups 2–4 had a SurR nucleotide binding sequence. All but one of the Group 4 *fhl* operons have a gene encoding for a TetR/AcrR family transcriptional regulator that is ∼350 nucleotides upstream of and transcribed in the same direction as the *fhl* operon (Supplementary Table 2). TetR/AcrR family transcriptional regulators are one-component systems where a single protein contains both a sensory domain and a DNA-binding domain (Cuthbertson and Nodwell, 2013). They are widely associated with antibiotic resistance and the regulation of genes encoding small molecule exporters and are usually encoded alongside target operons (Colclough et al., 2019). In *T. paralvinellae*, expression of the Group 4 *fhl* operon decreased when cells were grown on formate relative to growth on maltose or peptides (Topçuoğlu et al., 2018). The mechanism for regulation of Group 2–5 formate dehydrogenase-related genes is unknown.

## 9. Conclusion

Formate and H_2_ are linked both in hydrothermal vent environments and in the metabolisms of extremely thermophilic *Methanococci* and *Thermococci*. *Methanococci* prefer H_2_ oxidation to formate oxidation but appear to switch to the latter when H_2_ is limiting. Similarly, *Thermococci* appear to prefer H_2_ production to formate production but switch to the latter when H_2_ is excessive and inhibitory. H_2_ is typically far more abundant than formate in hydrothermal vent fluids suggesting that in high H_2_ environments formate is unlikely to be used by *Methanococci* and *Methanopyri* for growth. However, in hydrothermal environments that are very low H_2_ environments but rich in organic compounds, *Thermococci* may produce H_2_ and formate that are then used to support the growth of extremely thermophilic methanogens. Understanding where, when, and how formate is used by extreme thermophiles in nature is largely unknown and an area of future research. Furthermore, our understanding of transcriptional regulation of *fhl* and *nfd* in *Thermococci* is nascent. A key question is if and how formate influences gene expression, especially in concert with SurR regulation of hydrogenases and sulfur responsive genes.

## Author contributions

JH and HS contributed to the conceptualization, original draft preparation, review, and editing of the manuscript. JH conducted bioinformatic analyses and data compilation. Both authors read and agreed to the published version of the manuscript.

## References

[B1] Adams M. W. W. Holden J. F. Lal Menon A. Schut G. J. Grunden A. M. Hou C. (2001). Key role for sulfur in peptide metabolism and in regulation of three hydrogenases in the hyperthermophilic archaeon *Pyrococcus furiosus*. *J. Bacteriol.* 183 716–724. 10.1128/JB.183.2.716-724.2001 11133967 PMC94929

[B2] Anderson R. E. Beltrán M. T. Hallam S. J. Baross J. A. (2013). Microbial community structure across fluid gradients in the Juan de Fuca ridge hydrothermal system. *FEMS Microbiol. Ecol.* 83 324–339. 10.1111/j.1574-6941.2012.01478.x 22928928

[B3] Atomi H. Fukui T. Kanai T. Morikawa M. Imanaka T. (2004). Description of *Thermococcus kodakaraensis* sp. nov., a well studied hyperthermophilic archaeon previously reported as *Pyrococcus* sp. KOD1. *Archaea* 1 263–267. 15810436 10.1155/2004/204953 PMC2685570

[B4] Bae S. S. Kim T. W. Lee H. S. Kwon K. K. Kim Y. J. Kim M.-S. (2012). H2 production from CO, formate, or starch using the hyperthermophilic archaeon, *Thermococcus onnurineus*. *Biotechnol. Lett.* 34 75–79. 10.1007/s10529-011-0732-3 21898132

[B5] Bae S. S. Kim Y. J. Yang S. H. Lim J. K. Jeon J. H. Lee H. S. (2006). *Thermococcus onnurineus* sp. nov., a hyperthermophilic archaeon isolated from a deep-sea hydrothermal vent area at the pacmanus Field. *J. Microbiol. Biotechnol.* 16 1826–1831.

[B6] Bar-On Y. M. Phillips R. Milo R. (2018). The biomass distribution on Earth. *Proc. Natl. Acad. Sci. U.S.A.* 115 6506–6511. 10.1073/pnas.1711842115 29784790 PMC6016768

[B7] Baumberger T. Früh-Green G. L. Thorseth I. H. Lilley M. D. Hamelin C. Bernasconi S. M. (2016). Fluid composition of the sediment-influenced loki’s castle vent field at the ultra-slow spreading arctic mid-ocean ridge. *Geochim. Cosmochim. Acta* 187 156–178. 10.1016/j.gca.2016.05.017

[B8] Belay N. Sparling R. Daniels L. (1986). Relationship of formate to growth and methanogenesis by *Methanococcus thermolithotrophicus*. *Appl. Environ. Microbiol.* 52 1080–1085. 10.1128/aem.52.5.1080-1085.1986 3098165 PMC239176

[B9] Belkin S. Wirsen C. O. Jannasch H. W. (1985). Biological and abiological sulfur reduction at high temperatures. *Appl. Environ. Microbiol.* 49 1057–1061. 10.1128/aem.49.5.1057-1061.1985 16346781 PMC238504

[B10] Berndt M. E. Allen D. E. Seyfried W. E. Jr. (1996). Reduction of CO2 during serpentinization of olivine at 300°C and 500 bar. *Geology* 24 351–354. 10.1130/0091-76131996024<0351:ROCDSO<2.3.CO;2

[B11] Birrien J.-L. Zeng X. Jebbar M. Cambon-Bonavita M.-A. Quérellou J. Oger P. (2011). *Pyrococcus yayanosii* sp. nov., an obligate piezophilic hyperthermophilic archaeon isolated from a deep-sea hydrothermal vent. *Int. J. Syst. Evol. Microbiol.* 61 2827–2831. 10.1099/ijs.0.024653-0 21239564

[B12] Bonch-Osmolovskaya E. A. Stetter K. O. (1991). Interspecies hydrogen transfer in cocultures of thermophilic Archaea. *System. Appl. Microbiol.* 14 205–208.

[B13] Boyd E. S. Schut G. J. Adams M. W. W. Peters J. W. (2014). Hydrogen metabolism and the evolution of biological respiration. *Microbe* 9 361–367.

[B14] Brazelton W. J. McGonigle J. M. Motamedi S. Pendleton H. L. Twing K. I. Miller B. C. (2022). Metabolic strategies shared by basement residents of the lost city hydrothermal field. *Appl. Environ. Microbiol.* 88:e0092922. 10.1128/aem.00929-22 35950875 PMC9469722

[B15] Brazelton W. J. Schrenk M. O. Kelley D. S. Baross J. A. (2006). Methane- and sulfur-metabolizing microbial communities dominate the lost city hydrothermal field ecosystem. *Appl. Environ. Microbiol.* 72 6257–6270. 10.1128/AEM.00574-06 16957253 PMC1563643

[B16] Brown A. M. Hoopes S. L. White R. H. Sarisky C. A. (2011). Purine biosynthesis in archaea: Variations on a theme. *Biol. Direct* 6:63. 10.1186/1745-6150-6-63 22168471 PMC3261824

[B17] Burggraf S. Fricke H. Neuner A. Kristjansson J. Rouvier P. Mandelco L. (1990). *Methanococcus igneus* sp. nov., a novel hyperthermophilic methanogen from a shallow submarine hydrothermal system. *System. Appl. Microbiol.* 13 263–269. 10.1016/s0723-2020(11)80197-9 11538305

[B18] Callac N. Oger P. Lesongeur F. Rattray J. E. Vannier P. Michoud G. (2016). *Pyrococcus kukulkanii* sp. nov., a hyperthermophilic, piezophilic archaeon isolated from a deep-sea hydrothermal vent. *Int. J. Syst. Evol. Microbiol.* 66 3142–3149. 10.1099/ijsem.0.001160 27189596

[B19] Canganella F. Jones W. J. Gambacorta A. Antranikian G. (1998). *Thermococcus guaymasensis* sp. nov. and *Thermococcus aggregans* sp. nov., two novel thermophilic archaea isolated from the Guaymas basin hydrothermal vent site. *Int. J. Syst. Bacteriol.* 48 1181–1185. 10.1099/00207713-48-4-1181 9828419

[B20] Colclough A. L. Scadden J. Blair J. M. A. (2019). TetR-family transcription factors in gram-negative bacteria: Conservation, variation and implications for efflux-mediated antimicrobial resistance. *BMC Genomics* 20:731. 10.1186/s12864-019-6075-5 31606035 PMC6790063

[B21] Costa K. C. Wong P. M. Wang T. Lie T. J. Dodsworth J. A. Swanson I. (2010). Protein complexing in a methanogen suggests electron bifurcation and electron delivery from formate to heterodisulfide reductase. *Proc. Natl. Acad. Sci. U.S.A.* 107 11050–11055. 10.1073/pnas.1003653107 20534465 PMC2890747

[B22] Costa K. C. Yoon S. H. Pan M. Burn J. A. Baliga N. S. Leigh J. A. (2013). Effects of H2 and formate on growth yield and regulation of methanogenesis in *Methanococcus maripaludis*. *J. Bacteriol.* 195 1456–1462. 10.1128/JB.02141-12 23335420 PMC3624518

[B23] Courtine D. Vince E. Maignien L. Philippon X. Gayet N. Shao Z. (2021). *Thermococcus camini* sp. nov., a hyperthermophilic and piezophilic archaeon isolated from a deep-sea hydrothermal vent at the mid-atlantic ridge. *Int. J. Syst. Evol. Microbiol.* 71:004853. 10.1099/ijsem.0.004853 34236955

[B24] Cuthbertson L. Nodwell J. R. (2013). The TETR family of regulators. *Microbiol. Mol. Biol. Rev.* 77 440–475. 10.1128/MMBR.00018-13 24006471 PMC3811609

[B25] Dalmasso C. Oger P. Seva G. Courtine D. L’Harison S. Garlaschelli A. (2016). *Thermococcus piezophilus* sp. nov., a novel hyperthermophilic and piezophilic archaeon with a broad pressure range for growth, isolated from a deepest hydrothermal vent at the mid-Cayman rise. *Syst. Appl. Microbiol.* 39 440–444. 10.1016/j.syapm.2016.08.003 27638197

[B26] Ding K. Seyfried W. E. Jr. Zhang Z. Tivey M. K. Von Damm K. L. Bradley A. M. (2005). The in situ pH of hydrothermal fluids at mid-ocean ridges. *Earth Planet. Sci. Lett.* 237 167–174. 10.1016/j.epsl.2005.04.041

[B27] Duffaud G. D. d’Hennezel O. B. Peek A. S. Reysenbach A.-L. Kelly R. M. (1998). Isolation and characterization of *Thermococcus barossii*, sp. nov., a hyperthermophilic archaeon isolated from a hydrothermal vent flange formation. *System. Appl. Microbiol.* 21 40–49. 9741109 10.1016/S0723-2020(98)80007-6

[B28] Erauso G. Reysenbach A.-L. Godfroy A. Meunier J. R. Crump B. Partensky F. (1993). *Pyrococcus abyssi* sp. nov., a new hyperthermophilic archaeon isolated from a deep-sea hydrothermalvent. *Arch. Microbiol.* 160 338–349.

[B29] Fiala G. Stetter K. O. (1986). *Pyrococcus furiosus* sp. nov. Represents a novel genus of marine heterotrophic archaebacteria growing optimally at 100°C. *Arch. Microbiol.* 145 56–61.

[B30] Fleming H.-C. Wuertz S. (2019). Bacteria and archaea on earth and their abundance in biofilms. *Nat. Rev. Microbiol.* 17 247–260. 10.1038/s41579-019-0158-9 30760902

[B31] Flores G. E. Campbell J. H. Kirshtein J. D. Meneghin J. Podar M. Steinberg J. I. (2011). Microbial community structure of hydrothermal deposits from geochemically different vent fields along the mid-atlantic ridge. *Environ. Microbiol.* 13 2158–2171. 10.1111/j.1462-2920.2011.02463.x 21418499

[B32] Flores G. E. Shakya M. Meneghin J. Yang Z. K. Seewald J. S. Wheat C. G. (2012). Inter-field variability in the microbial communities of hydrothermal vent deposits from a back-arc basin. *Geobiology* 10 333–346. 10.1111/j.1472-4669.2012.00325.x 22443386

[B33] Fortunato C. S. Larson B. Butterfield D. A. Huber J. A. (2018). Spatially distinct, temporally stable microbial populations mediate biogeochemical cycling at and below the seafloor in hydrothermal vent fluids. *Environ. Microbiol.* 20 769–784. 10.1111/1462-2920.14011 29205750

[B34] Gallant R. M. Von Damm K. L. (2006). Geochemical controls on hydrothermal fluids from the Kairei and Edmond vent fields, 23°-25°S, central Indian ridge. *Geochem. Geophys. Geosyst.* 7:Q06018. 10.1029/2005GC001067

[B35] Gamo T. Masuda H. Yamanaka T. Okamura K. Ishibashi J. Nakayama E. (2004). Discovery of a new hydrothermal venting site in the southernmost Mariana Arc: Al-rich hydrothermal plumes and white smoler activity associated with biogenic methane. *Geochem. J.* 38 527–534. 10.2343/geochemj.38.527

[B36] González J. M. Kato C. Horikoshi K. (1995). *Thermococcus peptonophilus* sp. nov., a fast-growing, extremely thermophilic archaebacterium isolated from deep-sea hydrothermal vents. *Arch. Microbiol.* 164 159–164. 7545383

[B37] González J. M. Masuchi Y. Robb F. T. Ammerman J. W. Maeder D. L. Yanagibayashi M. (1998). *Pyrococcus horikoshii* sp. nov., a hyperthermophilic archaeon isolated from a hydrothermal vent at the okinawa trough. *Extremophiles* 2 123–130. 10.1007/s007920050051 9672687

[B38] Gorlas A. Croce O. Oberto J. Gauliard E. Forterre P. Marguet E. (2014). *Thermococcus nautili* sp. nov., a hyperthermophilic archaeon isolated from a hydrothermal deep-sea vent. *Int. J. Syst. Evol. Microbiol.* 64 1802–1810. 10.1099/ijs.0.060376-0 24556637

[B39] Greening C. Biswas A. Carere C. R. Jackson C. J. Taylor M. C. Stott M. B. (2016). Genomic and metagenomic surveys of hydrogenase distribution indicate H2 is a widely utilized energy source for microbial growth and survival. *ISME J.* 10 761–777. 10.1038/ismej.2015.153 26405831 PMC4817680

[B40] Grote R. Li L. Tamaoka J. Kato C. Horikoshi K. Antranikian G. (1999). *Thermococcus siculi* sp. nov., a novel hyperthermophilic archaeon isolated from a deep-sea hydrothermal vent at the Mid-Okinawa Trough. *Extremophiles* 3 55–62. 10086845 10.1007/s007920050099

[B41] Han Y. Gonnella G. Adam N. Schippers A. Burkhardt L. Kurtz S. (2018). Hydrothermal chimneys host habitat-specific microbial communities: Analogues for studying the possible impact of mining seafloor massive sulfide deposits. *Sci. Rep.* 8:10386. 10.1038/s41598-018-28613-5 29991752 PMC6039533

[B42] Hendrickson E. L. Leigh J. A. (2008). Roles of coenzyme F420-reducing hydrogenases and hydrogen- and F420-dependent methlenetetrahydromethanopterin dehydrogenases in reduction of F420 and production of hydrogen during methanogenesis. *J. Bacteriol.* 190 4818–4821. 10.1128/JB.00255-08 18487331 PMC2447022

[B43] Hensley S. A. Moreira E. Holden J. F. (2016). Hydrogen production and enzyme activities in the hyperthermophile *Thermococcus paralvinellae* grown on maltose, tryptone, and agricultural waste. *Front. Microbiol.* 7:167. 10.3389/fmicb.2016.00167 26941713 PMC4762990

[B44] Holden J. F. Summit M. Baross J. A. (1998). Thermophilic and hyperthermophilic microorganisms in 3-30°C hydrothermal fluids following a deep-sea volcanic eruption. *FEMS Microbiol. Ecol.* 25 33–41. 10.1111/j.1574-6941.1998.tb00458.x

[B45] Holden J. F. Takai K. Summit M. Bolton S. Zyskowski J. Baross J. A. (2001). Diversity among three novel groups of hyperthermophilic deep-sea *Thermococcus* species from three sites in the Northeastern Pacific ocean. *FEMS Microbiol. Ecol.* 36 51–60. 11377773 10.1111/j.1574-6941.2001.tb00825.x

[B46] Horita J. Berndt M. E. (1999). Abiogenic methane formation and isotopic fractionation under hydrothermal conditions. *Science* 285 1055–1057. 10.1126/science.285.5430.1055 10446049

[B47] Hou J. Sievert S. M. Wang Y. Seewald J. S. Natarajan V. P. Wang F. (2020). Microbial succession during the transition from active to inactive stages of deep-sea hydrothermal vent sulfide chimneys. *Microbiome* 8:102. 10.1186/s40168-020-00851-8 32605604 PMC7329443

[B48] Huber H. Thomm M. König H. Thies G. Stetter K. O. (1982). *Methanococcus thermolithotrophicus*, a novel thermophilic lithotrophic methanogen. *Arch. Microbiol.* 132 47–50. 10.1007/BF00690816

[B49] Huber J. A. Butterfield D. A. Baross J. A. (2002). Temporal changes in archaeal diversity and chemistry in a mid-ocean ridge subseafloor habitat. *Appl. Environ. Microbiol.* 68 1585–1594. 10.1128/AEM.68.4.1585-1594.2002 11916672 PMC123862

[B50] Huber R. Stöhr J. Hohenhaus S. Rachel R. Burggraf S. Jannasch H. W. (1995). *Thermococcus chitonophagus* sp. nov., a novel, chitin-degrading, hyperthermophilic archaeum from a deep-sea hydrothermal vent environment. *Arch. Microbiol.* 164 255–264.

[B51] Hudson R. de Graaf R. Strandoo Rodin M. Ohno A. Lane N. McGlynn S. E. (2020). CO2 reduction driven by a pH gradient. *Proc. Natl. Acad. Sci. U.S.A.* 117 22873–22879. 10.1073/pnas.2002659117 32900930 PMC7502746

[B52] Jaeschke A. Jørgensen S. L. Bernasconi S. M. Pedersen R. B. Thorseth I. H. Früh-Green G. L. (2012). Microbial diversity of loki’s castle black smokers at the arctic mid-ocean ridge. *Geobiology* 10 548–561. 10.1111/gbi.12009 23006788

[B53] Jeanthon C. L’Haridon S. Reysenbach A. L. Corre E. Vernet M. Messner P. (1999). *Methanococcus vulcanius* sp. nov., a novel hyperthermophilic methanogen isolated from east pacific rise, and identification of *Methanococcus* sp. DSM 4213T as *Methanococcus fervens* sp. nov. *Int. J. Syst. Microbiol.* 49 583–589. 10.1099/00207713-49-2-583 10319479

[B54] Jeanthon C. L’Haridon S. Reysenbach A. L. Vernet M. Messner P. Sleytr U. B. (1998). *Methanococcus infernus* sp. nov., a novel hyperthermophilic lithotrophic methanogen isolated from a deep-sea hydrothermal vent. *Int. J. Syst. Bacteriol.* 48 913–919. 10.1099/00207713-48-3-913 9734046

[B55] Jolivet E. Corre E. L’Harison S. Forterre P. Prieur D. (2004). *Thermococcus marinus* sp. nov. and *Thermococcus radiotolerans* sp. nov., two hyperthermophilic archaea from deep-sea hydrothermal vents that resist ionizing radiation. *Extremophiles* 8 219–227. 10.1007/s00792-004-0380-9 14991422

[B56] Jolivet E. L’Haridon S. Corre E. Forterre P. Prieur D. (2003). *Thermococcus gammatolerans* sp. nov., a hyperthermophilic archaeon from a deep-sea hydrothermal vent that resists ionizing radiation. *Int. J. Syst. Evol. Microbiol.* 53 847–851. 10.1099/ijs.0.02503-0 12807211

[B57] Jones D. T. Taylor W. R. Thornton J. M. (1992). The rapid generation of mutation data matrices from protein sequences. *Comput. Appl. Biosci.* 8 275–282. 1633570 10.1093/bioinformatics/8.3.275

[B58] Jones W. J. Paynter M. J. B. Gupta R. (1983b). Characterization of *Methanococcus maripaludis* sp. nov., a new methanogen isolated from salt marsh sediment. *Arch. Microbiol.* 135 91–97. 10.1007/BF00408015

[B59] Jones W. J. Leigh J. A. Mayer F. Woese C. R. Wolfe R. S. (1983a). *Methanococcus jannaschii* sp. nov., an extremely thermophilic methanogen from a submarine hydrothermal vent. *Arch. Microbiol.* 136 254–261. 10.1007/BF00425213

[B60] Jung H.-C. Lim J. K. Yang T.-J. Kang S. G. Lee H. S. (2020). Direct electron transfer between the frhAGB-encoded hydrogenase and thioredoxin reductase in the nonmethanogenic archaeon *Thermococcus onnurineus* NA1. *Appl. Environ. Microbiol.* 86 e2630–e2619. 10.1128/AEM.02630-19 31924613 PMC7054103

[B61] Kaster A.-K. Moll J. Parey K. Thauer R. K. (2011). Coupling of ferredoxin and heterodisulfide reduction via electron bifurcation in hydrogenotrophic methanogenic archaea. *Proc. Natl. Acad. Sci. U.S.A.* 108 2981–2986. 10.1073/pnas.1016761108 21262829 PMC3041090

[B62] Kim Y. J. Lee H. S. Kim E. S. Bae S. S. Lim J. K. Matsumi R. (2010). Formate-driven growth coupled with H2 production. *Nature* 467 352–355. 10.1038/nature09375 20844539

[B63] Klein F. Tarnas J. D. Bach W. (2020). Abiotic sources of molecular hydrogen on Earth. *Elements* 16 19–24. 10.2138/gselements.16.1.19

[B64] Kobayashi T. Kwak Y. S. Akiba T. Kudo T. Horikoshi K. (1994). *Thermococcus profundus* sp. nov., a new hyperthermophilic archaeon isolated from a deep-sea hydrothermal vent. *Syst. Appl. Microbiol.* 17 232–236.

[B65] Konn C. Donval J. P. Guyader V. Germain Y. Alix A.-S. Roussel E. (2022). Extending the dataset of fluid geochemistry of the menez Gwen, lucky strike, rainbow, tag and snake pit hydrothermal vent fields: Investigation of temporal stability and organic contribution. *Deep Sea Res.* 179:103630. 10.1016/j.dsr.2021.103630

[B66] Kormas K. A. Tivey M. K. Von Damm K. Teske A. (2006). Bacterial and archaeal phylotypes associated with distinct mineralogical layers of a white smoker spire from a deep-sea hydrothermal vent site (9°N, east pacific rise). *Environ. Microbiol.* 8 909–920. 10.1111/j.1462-2920.2005.00978.x 16623747

[B67] Kumagai H. Nakamura K. Toki T. Morishita T. Okino K. Ishibashi J.-I. (2008). Geological background of the Kairei and Edmond hydrothermal fields along the central Indian ridge: Implications of their vent fluids’ distinct chemistry. *Geofluids* 8 239–251. 10.1111/j.1468-8123.2008.00223.x

[B68] Kurr M. Huber R. König H. Jannasch H. W. Fricke H. Trincone A. (1991). *Methanopyrus kandleri*, gen. and sp. nov., represents a novel group of hyperthermophilic methanogens, growing at 110°C. *Arch. Microbiol.* 156 239–247. 10.1007/BF00262992

[B69] Lang S. Q. Butterfield D. A. Schulte M. Kelley D. S. Lilley M. D. (2010). Elevated concentrations of formate, acetate and dissolved organic carbon found at the lost city hydrothermal field. *Geochim. Cosmochim. Acta* 74 941–952. 10.1016/j.gca.2009.10.045

[B70] Lang S. Q. Früh-Green G. L. Bernasconi S. M. Brazelton W. J. Schrenk M. O. McGonigle J. M. (2018). Deeply-sourced formate fuels sulfate reducers but not methanogens at lost city hydrothermal field. *Sci. Rep.* 8:755. 10.1038/s41598-017-19002-5 29335466 PMC5768773

[B71] Lang S. Q. Früh-Green G. L. Bernasconi S. M. Lilley M. D. Proskurowski G. Méhay S. (2012). Microbial utilization of abiogenic carbon and hydrogen in a serpentinite-hosted system. *Geochim. Cosmochim. Acta* 92 82–99. 10.1016/j/gca.2012.06.006 29786478

[B72] Le Guellec S. Leroy E. Courtine D. Godfroy A. Roussel E. G. (2021). H2-dependent formate production by hyperthermophilic thermococcales: An alternative to sulfur reduction for reducing-equivalents disposal. *ISME J.* 15 3423–3436. 10.1038/s41396-021-01020-x 34088977 PMC8630068

[B73] Lie T. J. Costa K. C. Lupa B. Korpole S. Whitman W. B. Leigh J. A. (2012). Essential anaplerotic role for the energy-converting hydrogenase eha in hydrogenotrophic methanogenesis. *Proc. Natl. Acad. Sci. U.S.A.* 109 15473–15478. 10.1073/pnas.1208779109 22872868 PMC3458328

[B74] Lilley M. D. Butterfield D. A. Lupton J. E. Olson E. J. (2003). Magmatic events can produce rapid changes in hydrothermal vent chemistry. *Nature* 422 878–881. 10.1038/nature01569 12712202

[B75] Lim J. K. Jung H.-C. Kang S. G. Lee H. S. (2017). Redox regulation of SurR by protein disulfide oxidoreductase in *Thermococcus onnurineus* NA1. *Extremophiles* 21 491–498. 10.1007/s00792-017-0919-1 28251348

[B76] Lim J. K. Kim Y. J. Yang J.-A. Namirimu T. Yang S.-H. Park M.-J. (2020). *Thermococcus indicus* sp. nov., a Fe(III)-reducing hyperthermophilic archaeon isolated from the onnuri vent field of the central Indian ocean ridge. *J. Microbiol.* 58 260–267. 10.1007/s12275-020-9424-9 32239454

[B77] Lim J. K. Mayer F. Kang S. G. Müller V. (2014). Energy conservation by oxidation of formate to carbon dioxide and hydrogen via a sodium ion current in a hyperthermophilic archaeon. *Proc. Natl. Acad. Sci. U.S.A.* 111 11497–11502. 10.1073/pnas.1407056111 25049407 PMC4128143

[B78] Lin T. J. Ver Eecke H. C. Breves E. A. Dyar M. D. Jamieson J. W. Hannington M. D. (2016). Linkages between mineralogy, fluid chemistry, and microbial communities within hydrothermal chimneys from the endeavour segment, Juan de Fuca ridge. *Geochem. Geophys. Geosyst.* 17 300–323. 10.1002/2015GC006091 30123099 PMC6094386

[B79] Lipscomb G. L. Keese A. M. Cowart D. M. Schut G. J. Thomm M. Adams M. W. W. (2009). SurR: A transcriptional activator and repressor controlling hydrogen and elemental sulfur metabolism in *Pyrococcus furiosus*. *Mol. Microbiol.* 71 332–349. 10.1111/j.1365-2958.2008.06525.x 19017274 PMC2745277

[B80] Lipscomb G. L. Schut G. J. Scott R. A. Adams M. W. W. (2017). SurR is a master regulator of the primary electron flow pathways in the order thermococcales. *Mol. Microbiol.* 104 869–881. 10.1111/mmi.13668 28295726

[B81] Lupa B. Hendrickson E. L. Leigh J. A. Whitman W. B. (2008). Formate-dependent H2 production by the mesophilic methanogen *Methanococcus maripaludis*. *Appl. Environ. Microbiol.* 74 6584–6590. 10.1128/AEM.01455-08 18791018 PMC2576700

[B82] Marteinsson V. T. Birrien J.-L. Reysenbach A.-L. Vernet M. Marie D. Gambacorta A. (1999). *Thermococcus barophilus* sp. nov., a new barophilic and hyperthermophilic archaeon isolated under high hydrostatic pressure from a deep-sea hydrothermal vent. *Int. J. Syst. Bacteriol.* 49 351–359. 10319455 10.1099/00207713-49-2-351

[B83] McCliment E. A. Voglesonger K. M. O’Day P. A. Dunn E. E. Holloway J. R. Cary S. C. (2006). Colonization of nascent, deep-sea hydrothermal vents by a novel archaeal and nanoarchaeal assemblage. *Environ. Microbiol.* 8 114–125. 10.1111/j.1462-2920.2005.00874.x 16343327

[B84] McCollom T. M. Seewald J. S. (2001). A reassessment of the potential for reduction of dissolved CO2 to hydrocarbons during serpentinization of olivine. *Geochim. Cosmochim. Acta* 65 3769–3778. 10.1016/S0016-7037(01)00655-X

[B85] McCollom T. M. Seewald J. S. (2003). Experimental constraints on the hydrothermal reactivity of organic acids and acid anions: I. Formic acid and formate. *Geochim. Cosmochim. Acta* 67 3625–3644. 10.1016/S0016-7037(03)00136-4

[B86] McDermott J. M. Seewald J. S. German C. R. Sylva S. P. (2015). Pathways for abiotic organic synthesis at submarine hydrothermal fields. *Proc. Natl. Acad. Sci. U.S.A.* 112 7668–7672. 10.1073/pnas.1506295112 26056279 PMC4485091

[B87] McDermott J. M. Sylva S. P. Ono S. German C. R. Seewald J. S. (2018). Geochemistry of fluids from Earth’s deepest ridge-crest hot-springs: Piccard hydrothermal field, mid-Cayman rise. *Geochim. Cosmochim. Acta* 228 95–118. 10.1016/j.gca.2018.01.021

[B88] McGonigle J. M. Lang S. Q. Brazelton W. J. (2020). Genomic evidence for formate metabolism by chloroflexi as the key to unlocking deep carbon in lost city microbial ecosystems. *Appl. Environ. Microbiol.* 86 e2583–e2519. 10.1128/AEM.02583-19 32033949 PMC7117926

[B89] Meyer J. L. Akerman N. H. Proskurowski G. Huber J. A. (2013). Microbiological characterization of post-eruption “snowblower” vents at axial seamount, Juan de Fuca ridge. *Front. Microbiol.* 4:153. 10.3389/fmicb.2013.00153 23785361 PMC3683637

[B90] Miroshnichenko M. L. Gongadze G. M. Rainey F. A. Kostyukova A. S. Lysenko A. M. Chernyh N. A. (1998). *Thermococcus gorgonarius* sp. nov. and *Thermococcus pacificus* sp. nov: Heterotrophic extremely thermophilic archaea from New Zealand submarine hot vents. *Int. J. Syst. Bacteriol.* 48 23–29. 10.1099/00207713-48-1-23 9542072

[B91] Miroshnichenko M. L. Hippe H. Stackebrandt E. Kostrikina N. A. Chernyh N. A. Jeanthon C. (2001). Isolation and characterization of *Thermococcus sibiricus* sp. nov. From a western Siberia high-temperature oil reservoir. *Extremophiles* 5 85–91. 10.1007/s007920100175 11354459

[B92] Moon Y.-J. Kwon J. Yun S.-H. Lim H. L. Kim M.-S. Kang S. G. (2012). Proteome analyses of hydrogen-producing hyperthermophilic archaeon *Thermococcus onnurineus* NA1 in different one-carbon substrate culture conditions. *Mol. Cell. Proteomics* 11:M111.015420. 10.1074/mcp.M111.015420 22232491 PMC3433910

[B93] Nakagawa T. Takai K. Suzuki Y. Hirayama H. Konno U. Tsunogai U. (2006). Geomicrobiological exploration and characterization of a novel deep-sea hydrothermal system at the TOTO caldera in the Mariana volcanic Arc. *Environ. Microbiol.* 8 37–49. 10.1111/j.1462-2920.2005.00884.x 16343320

[B94] Niks D. Hille R. (2019). Molybdenum- and tungsten-containing formate dehydrogenases and formylmethanofuran dehydrogenases: Structure, mechanism, and cofactor insertion. *Protein Soc.* 28 111–122. 10.1002/pro.3498 30120799 PMC6295890

[B95] Nolling J. Reeve J. N. (1997). Growth- and substrate-dependent transcription of the formate dehydrogenase (fdhCAB) operon in *Methanobacterium thermoformicicum* Z-245. *J. Bacteriol.* 179 899–908. 10.1128/jb.179.3.899-908.1997 9006048 PMC178775

[B96] Ownby K. Xu H. White R. H. (2005). A *Methanocaldococcus jannaschii* archaeal signature gene encodes for a 5-formaminoimidazole-4-carboxamide-1-β-D-ribofuranosyl 5’-monophosphate synthetase. *J. Biol. Chem.* 280 10881–10887. 10.1074/jbc.M413937200 15623504

[B97] Pagé A. Tivey M. K. Stakes D. S. Reysenbach A.-L. (2008). Temporal and spatial archaeal colonization of hydrothermal vent deposits. *Environ. Microbiol.* 10 874–884. 10.1111/j.1462-2920.2007.01505.x 18201197

[B98] Perner M. Kuever J. Seifert R. Pape T. Koschinsky A. Schmidt K. (2007). The influence of ultramafic rocks on microbial communities at the logatchev hydrothermal field, located 15°N on the Mid-Atlantic Ridge. *FEMS Microbiol. Ecol.* 16 97–109. 10.1111/j.1574-6941.2007.00325.x 17506828

[B99] Pledger R. J. Baross J. A. (1989). Characterization of an extremely thermophilic archaebacterium from a black smoker polychaete (*Paralvinella* sp.) at the Juan de Fuca ridge. *System. Appl. Microbiol.* 12 249–256.

[B100] Porat I. Kim W. Hendrickson E. L. Xia Q. Zhang Y. Wang T. (2006). Disruption of the operon encoding Ehb hydrogenase limits anabolic CO2 assimilation in the archaeon *Methanococcus maripaludis*. *J. Bacteriol.* 188 1373–1380. 10.1128/JB.188.4.1373-1380.2006 16452419 PMC1367223

[B101] Reveillaud J. Reddington E. McDermott J. Algar C. Meyer J. L. Sylva S. (2016). Subseafloor microbial communities in hydrogen-rich vent fluids from hydrothermal systems along the mid-Cayman rise. *Environ. Microbiol.* 18 1970–1987. 10.1111/1462-2920.13173 26663423 PMC5021209

[B102] Reysenbach A.-L. St. John E. Meneghin J. Flores G. E. Podar M. Dombrowski N. (2020). Complex subsurface hydrothermal fluid mixing at a submarine arc volcano supports distinct and highly diverse microbial communities. *Proc. Natl. Acad. Sci. U.S.A.* 117 32627–32638. 10.1073/pnas.2019021117 33277434 PMC7768687

[B103] Sakai S. Takaki Y. Miyazaki M. Ogawara M. Yanagawa K. Miyazaki J. (2019). *Methanofervidicoccus abyssi* gen. nov., sp. nov., a hydrogenotrophic methanogen, isolated from a hydrothermal chimney in the mid-cayman spreading center, the Caribbean sea. *Int. J. Syst. Evol. Microbiol.* 69 1225–1230. 10.1099/ijsem.0.003297 30843780

[B104] Sapra R. Bagramyan K. Adams M. W. W. (2003). A simple energy-conserving system: Proton reduction coupled to proton translocation. *Proc. Natl. Acad. Sci. U.S.A.* 100 7545–7550. 10.1073/pnas.1331436100 12792025 PMC164623

[B105] Sattler C. Wolf S. Fersch J. Goetz S. Rother M. (2013). Random mutagenesis identifies factors involved in formate-dependent growth of the methanogenic archaeon *Methanococcus maripaludis*. *Mol. Genet. Genomics* 288 413–424. 10.1007/s00438-013-0756-6 23801407

[B106] Schink B. Montag D. Keller A. Müller N. (2017). Hydrogen or formate: Alternative key players in methanogenic degradation. *Environ. Microbiol. Rep.* 9 189–202. 10.1111/1758-2229.12524 28205388

[B107] Schrenk M. O. Kelley D. S. Bolton S. A. Baross J. A. (2004). Low archaeal diversity linked to subseafloor geochemical processes at the lost city hydrothermal field, mid-atlantic ridge. *Environ. Microbiol.* 6 1086–1095. 10.1111/j.1462-2920.2004.00650.x 15344934

[B108] Schut G. J. Bridger S. L. Adams M. W. W. (2007). Insights into the metabolism of elemental sulfur by the hyperthermophilic archaeon *Pyrococcus furiosus*: Characterization of a coenzyme A-dependent NAD(P)H sulfur oxidoreductase. *J. Bacteriol.* 189 4431–4441. 10.1128/JB.00031-07 17449625 PMC1913366

[B109] Schut G. J. Nixon W. J. Lipscomb G. L. Scott R. A. Adams M. W. W. (2012). Mutational analyses of the enzymes involved in the metabolism of hydrogen by the hyperthermophilic archaeon *Pyrococcus furiosus*. *Front. Microbiol.* 3:163. 10.3389/fmicb.2012.00163 22557999 PMC3341082

[B110] Schut G. J. Zhou J. Adams M. W. W. (2001). DNA microarray analysis of the hyperthermophilic archaeon *Pyrococcus furiosus*: Evidence for a new type of sulfur-reducing enzyme complex. *J. Bacteriol.* 183 7027–7036. 11717259 10.1128/JB.183.24.7027-7036.2001 PMC95549

[B111] Seewald J. S. Zolotov M. Y. McCollom T. (2006). Experimental investigation of single carbon compounds under hydrothermal conditions. *Geochim. Cosmochim. Acta* 70 446–460. 10.1016/j.gca.2005.09.002

[B112] Seewald J. Cruse A. Saccocia P. (2003). Aqueous volatiles in hydrothermal fluids from the main endeavour field, northern Juan de Fuca ridge: Temporal variability following earthquake activity. *Earth Planet. Sci. Lett.* 216 575–590. 10.1016/S0012-821X(03)00543-0

[B113] Shock E. L. (1990). Geochemical constraints on the origin of organic compounds in hydrothermal systems. *Orig. Life Evol. Biosph.* 20 331–367. 10.1007/BF01581580 11536667

[B114] Sokolova T. G. Jeanthon C. Kostrikina N. A. Chernyh N. A. Lebedinsky A. V. Stackebrandt E. (2004). The first evidence of anaerobic CO oxidation coupled with H2 production by a hyperthermophilic archaeon isolated from a deep-sea hydrothermal vent. *Extremophiles* 8 317–323. 10.1007/s00792-004-0389-0 15164268

[B115] Sprott G. D. Ekiel I. Patel G. B. (1993). Metabolic pathways in *Methanococcus jannaschii* and other methanogenic bacteria. *Appl. Environ. Microbiol.* 59 1092–1098. 10.1128/AEM.59.4.1092-1098.1993 16348909 PMC202243

[B116] Stetter K. O. (2006). History of discovery of the first hyperthermophiles. *Extremophiles* 10 357–362. 10.1007/s00792-006-0012-7 16941067

[B117] Stewart L. C. Algar C. K. Fortunato C. S. Larson B. I. Vallino J. J. Huber J. A. (2019). Fluid geochemistry, local hydrology, and metabolic activity define methanogen community size and composition in deep-sea hydrothermal vents. *ISME J.* 13 1711–1721. 10.1038/s41396-019-0382-3 30842565 PMC6776001

[B118] Takai K. Gamo T. Tsunogai U. Nakayama N. Hirayama H. Nealson K. H. (2004b). Geochemical and microbiological evidence for a hydrogen-based, hyperthermophilic subsurface lithoautotrophic microbial ecosystem (HyperSLiME) beneath an active deep-sea hydrothermal field. *Extremophiles* 8 269–282. 10.1007/s00792-004-0386-3 15309563

[B119] Takai K. Inoue A. Horikoshi K. (2002). *Methanothermococcus okinawensis* sp. nov., a thermophilic, methane-producing archaeon isolated from a Western Pacific deep-sea hydrothermal vent system. *Int. J. Syst. Evol. Microbiol.* 52 1089–1095. 10.1099/00207713-52-4-1089 12148612

[B120] Takai K. Nealson K. H. Horikoshi K. (2004a). *Methanotorris formicicus* sp. nov., a novel extremely thermophilic, methane-producing archaeon isolated from a black smoker chimney in the central Indian ridge. *Int. J. Syst. Evol. Microbiol.* 54 1095–1100. 10.1099/ijs.0.02887-0 15280275

[B121] Takai K. Nunoura T. Horikoshi K. Shibuya T. Nakamura K. Suzuki Y. (2009). Variability in microbial communities in black smoker chimneys at the NW caldera vent field, brothers volcano, kermadec Arc. *Geomicrobiol. J.* 26 252–269. 10.1080/01490450903304949

[B122] Takai K. Nunoura T. Ishibashi J. Lupton J. Suzuki R. Hamasaki H. (2008). Variability in the microbial communities and hydrothermal fluid chemistry at the newly discovered mariner hydrothermal field, southern Lau basin. *J. Geophys. Res.* 113:G02031. 10.1029/2007JG000636

[B123] Tamura K. Stecher G. Kumar S. (2021). MEGA11: Molecular evolutionary genetics analysis version 11. *Mol. Biol. Evol.* 38 3022–3027. 10.1093/molbev/msab120 33892491 PMC8233496

[B124] Thauer R. K. Kaster A.-K. Seedorf H. Buckel W. Hedderich R. (2008). Methanogenic archaea: Ecologically relevant differences in energy conservation. *Nat. Rev. Microbiol.* 6 579–591. 10.1038/nrmicro1931 18587410

[B125] Topçuoğlu B. D. Meydan C. Nguyen T. B. Lang S. Q. Holden J. F. (2019). Growth kinetics, carbon isotope fractionation, and gene expression in the hyperthermophile *Methanocaldococcus jannaschii* during hydrogen-limited growth and interspecies hydrogen transfer. *Appl. Environ. Microbiol.* 85 e180–e119. 10.1128/AEM.00180-19 30824444 PMC6495749

[B126] Topçuoğlu B. D. Meydan C. Orellana R. Holden J. F. (2018). Formate hydrogenlyase and formate secretion ameliorate H2 inhibition in the hyperthermophilic archaeon *Thermococcus paralvinellae*. *Environ. Microbiol.* 20 949–957. 10.1111/1462-2920.14022 29235714

[B127] Topçuoğlu B. D. Stewart L. C. Morrison H. G. Butterfield D. A. Huber J. A. Holden J. F. (2016). Hydrogen limitation and syntrophic growth among natural assemblages of thermophilic methanogens at deep-sea hydrothermal vents. *Front. Microbiol.* 7:1240. 10.3389/fmicb.2016.01240 27547206 PMC4974244

[B128] Valentine D. L. Chidthaisong A. Rice A. Reeburgh W. S. Tyler S. C. (2004). Carbon and hydrogen isotope fractionation by moderately thermophilic methanogens. *Geochim. Cosmochim. Acta* 68 1571–1590. 10.1016/j.gca.2003.10.012

[B129] Van Haaster D. J. Silva P. J. Hagedoorn P.-L. Jongejan J. A. Hagen W. R. (2008). Reinvestigation of the steady-state kinetics and physiological function of the soluble NiFe-hydrogenase I of *Pyrococcus furiosus*. *J. Bacteriol.* 190 1584–1587. 10.1128/JB.01562-07 18156274 PMC2258664

[B130] Ver Eecke H. C. Akerman N. H. Huber J. A. Butterfield D. A. Holden J. F. (2013). Growth kinetics and energetics of a deep-sea hyperthermophilic methanogen under varying environmental conditions. *Environ. Microbiol. Rep.* 5 665–671. 10.1111/1758-2229.12065 24115616

[B131] Ver Eecke H. C. Butterfield D. A. Huber J. A. Lilley M. D. Olson E. J. Roe K. K. (2012). Hydrogen-limited growth of hyperthermophilic methanogens at deep-sea hydrothermal vents. *Proc. Natl. Acad. Sci. U.S.A.* 109 13674–13679. 10.1073/pnas.1206632109 22869718 PMC3427048

[B132] Ver Eecke H. C. Kelley D. S. Holden J. F. (2009). Abundances of hyperthermophilic autotrophic Fe(III) oxide reducers and heterotrophs in hydrothermal sulfide chimneys of the Northeastern Pacific ocean. *Appl. Environ. Microbiol.* 75 242–245. 10.1128/AEM.01462-08 18978076 PMC2612199

[B133] Von Damm K. L. Lilley M. D. (2004). “Diffuse flow hydrothermal fluids from 9° 50’ N east pacific rise: Origin, evolution and biogeochemical controls,” in *The subseafloor biosphere at mid-ocean ridges*, eds Wilcock W. S. D. DeLong E. F. Kelley D. S. Cary S. C. (Washington, DC: American Geophysical Union Press), 245–268.

[B134] Von Damm K. L. Edmond J. M. Measures C. I. Grant B. (1985). Chemistry of submarine hydrothermal solutions at Guaymas basin, gulf of California. *Geochim. Cosmochim. Acta* 49 2221–2237. 10.1016/0016-7037(85)90223-6

[B135] White R. H. (1997). Purine biosynthesis in the domain archaea without folates or modified folates. *J. Bacteriol.* 179 3374–3377. 10.1128/jb.179.10.3374-3377.1997 9150241 PMC179124

[B136] Windman T. Zolotova N. Schwandner F. Shock E. L. (2007). Formate as an energy source for microbial metabolism in chemosynthetic zones of hydrothermal ecosystems. *Astrobiology* 7 873–890. 10.1089/ast.2007.0127 18163868

[B137] Wood G. E. Haydock A. K. Leigh J. A. (2003). Function and regulation of the formate dehydrogenase genes of the methanogenic archaeon *Methanococcus maripaludis*. *J. Bacteriol.* 185 2548–2554. 10.1128/JB.185.8.2548-2554.2003 12670979 PMC152622

[B138] Wu C.-H. Schut G. J. Poole F. II Haja D. K. Adams M. W. W. (2018). Characterization of membrane-bound sulfane reductase: A missing link in the evolution of modern day respiratory complexes. *J. Biol. Chem.* 293 16687–16696. 10.1074/jbc.RA118.005092 30181217 PMC6204914

[B139] Yang H. Lipscomb G. L. Keese A. M. Schut G. J. Thomm M. Adams M. W. W. (2010). SurR regulates hydrogen production in *Pyrococcus furiosus* by a sulfur-dependent redox switch. *Mol. Microbiol.* 77 1111–1122. 10.1111/j.1365-2958.2010.07275.x 20598080 PMC2975895

[B140] Yang J. Lee S. H. Ryu J. Y. Lee H. S. Kang S. G. (2022). A novel NADP-dependent formate dehydrogenase from the hyperthermophilic archaeon *Thermococcus onnurineus* NA1. *Front. Microbiol.* 13:844735. 10.3389/fmicb.2022.844735 35369452 PMC8965080

[B141] Zeng X. Zhang X. Jiang L. Alain K. Jebbar M. Shao Z. (2013). *Palaeococcus pacificus* sp. nov., an archaeon from deep-sea hydrothermal sediment. *Int. J. Syst. Evol. Microbiol.* 63 2155–2159. 10.1099/ijs.0.044487-0 23104364

[B142] Zhao H. Wood A. G. Widdel F. Bryant M. P. (1988). An extremely thermophilic *Methanococcus* from a deep-sea hydrothermal vent and its plasmid. *Arch. Microbiol.* 150 178–183. 10.1007/BF00425159

[B143] Zhao W. Zeng X. Xiao X. (2015). *Thermococcus eurythermalis* sp. nov., a conditional piezophilic, hyperthermophilic archaeon with a wide temperature range for growth, isolated from an oil-immersed chimney in the Guaymas basin. *Int. J. Syst. Evol. Microbiol.* 65 30–35. 10.1099/ijs.0.067942-0 25288278

[B144] Zillig W. Holz I. Janekovic D. Schäfer W. Reiter W. D. (1983). The archaebacterium *Thermococcus* celer represents, a novel genus within the thermophilic branch of the archaebacteria. *System. Appl. Microbiol.* 4 88–94. 10.1016/S0723-2020(83)80036-8 23196302

