# Supplementary material for: Formate and hydrogen in hydrothermal vents and their use by extremely thermophilic methanogens and heterotrophs
Source: Front Microbiol. 2023 Mar 6;14:1093018. doi: 10.3389/fmicb.2023.1093018 (PMC10025317; doi:10.3389/fmicb.2023.1093018)
Supplement: Supplementary file 3 [file Data_Sheet_1.PDF]

Supplementary Material

Formate and Hydrogen in Hydrothermal Vents and Their Use by Extremely Thermophilic Methanogens and Heterotrophs

James F. Holden[1]*, Harita Sistu[1]

[1]Department of Microbiology, University of Massachusetts, Amherst, Massachusetts, USA

*** Correspondence:** James F. Holden: jholden@umass.edu

1 Supplementary Methods

Identification of gene candidates

Thirty fully sequenced, non-draft *Thermococci* genomes, seven *Methanococci* genomes, and a *Methanopyrus kandleri* genome available in the National Center for Biotechnology Information (NCBI) genome database were considered. Three draft *Methanococci* genomes were also included to increase representation. The organisms studied and their genome accession numbers are listed in Tables 2 and 3.

Gene candidates were identified using the protein BLAST (BLASTp) search tool provided through the NCBI site https://blast.ncbi.nlm.nih.gov/Blast.cgi, with results limited to the selected genomes. Known enzymes from *Methanococcus maripaludis*, *Methanocaldococcus jannaschii*, *Pyrococcus furiosus*, and *Thermococcus onnurineus* (Table S1) were used as input sequences for the BLAST searches. Each gene of each putative operon was identified. Genes were considered part of an operon if they were syntenic and within 50 nucleotides of each other on the genome.

2 References

Brown, A. M., Hoopes, S. L., White, R. H., and Sarisky, C. A. (2011). Purine biosynthesis in archaea: variations on a theme. Biol. Direct 6:63. doi: 10.1186/1745-6150-6-63

Hendrickson, E. L., and Leigh, J. A. (2008). Roles of coenzyme F_{420}-reducing hydrogenases and hydrogen- and F_{420}-dependent methlenetetrahydromethanopterin dehydrogenases in reduction of F_{420} and production of hydrogen during methanogenesis. J. Bacteriol. 190, 4818-4821. doi: 10.1128/JB.00255-08

Jung, H.-C., Lim, J. K., Yang, T.-J., Kang, S. G., and Lee, H. S. (2020). Direct electron transfer between the *frhAGB*-encoded hydrogenase and thioredoxin reductase in the nonmethanogenic archaeon *Thermococcus onnurineus* NA1. Appl. Environ. Microbiol. 86:e02630-19. doi: 10.1128/AEM.02630-19

Kaster, A.-K., Moll, J., Parey, K., and Thauer, R. K. (2011). Coupling of ferredoxin and heterodisulfide reduction via electron bifurcation in hydrogenotrophic methanogenic archaea. Proc. Natl. Acad. Sci. USA 108, 2981-2986. doi: 10.1073/pnas.1016761108

Lie, T. J., Costa, K. C., Lupa, B., Korpole, S., Whitman, W. B., and Leigh, J. A. (2012). Essential anaplerotic role for the energy-converting hydrogenase Eha in hydrogenotrophic methanogenesis. Proc. Natl. Acad. Sci. USA 109, 15473-15478. doi: 10.1073/pnas.1208779109

Moon, Y.-J., Kwon, J., Yun, S.-H., Lim, H. L. Kim, M.-S., Kang, S. G., Lee, J.-H., Choi, J.-S., Kim, S. I., and Chung, Y.-H. (2012). Proteome analyses of hydrogen-producing hyperthermophilic archaeon *Thermococcus onnurineus* NA1 in different one-carbon substrate culture conditions. Mol. Cell. Proteomics 11:M111.015420. doi: 10.1074/mcp.M111.015420

Ownby, K., Xu, H., and White, R. H. (2005). A *Methanocaldococcus jannaschii* archaeal signature gene encodes for a 5-formaminoimidazole-4-carboxamide-1-β-D-ribofuranosyl 5'-monophosphate synthetase. J. Biol. Chem. 280, 10,881-10,887. doi: 10.1074/jbc.M413937200

Sapra, R., Bagramyan, K., and Adams, M. W. W. (2003). A simple energy-conserving system: proton reduction coupled to proton translocation. Proc. Natl. Acad. Sci. USA 100, 7545-7550. doi: 10.1073/pnas.1331436100

Sattler, C., Wolf, S., Fersch, J., Goetz, S., and Rother, M. (2013). Random mutagenesis identifies factors involved in formate-dependent growth of the methanogenic archaeon *Methanococcus maripaludis*. Mol. Genet. Genomics 288, 413-424. doi: 10.1007/s00438-013-0756-6

Van Haaster, D. J., Silva, P. J., Hagedoorn, P.-L., Jongejan, J. A., and Hagen, W. R. (2008). Reinvestigation of the steady-state kinetics and physiological function of the soluble NiFe-hydrogenase I of *Pyrococcus furiosus*. J. Bacteriol. 190, 1584-1587. doi: 10.1128/JB.01562-07

Wood, G. E., Haydock, A. K., and Leigh, J. A. (2003). Function and regulation of the formate dehydrogenase genes of the methanogenic archaeon *Methanococcus maripaludis*. J. Bacteriol. 185, 2548-2554. doi: 10.1128/JB.185.8.2548–2554.2003

Yang, J., Lee, S. H., Ryu, J. Y., Lee, H. S., and Kang, S. G. (2022). A novel NADP-dependent formate dehydrogenase from the hyperthermophilic archaeon *Thermococcus onnurineus* NA1. Front. Microbiol. 13:844735. doi: 10.3389/fmicb.2022.844735

Table S1. Proteins used to search for gene candidates in *Methanococci*, *Methanopyri*, and *Thermococci* whole genome sequences using the NCBI protein BLAST (BLASTp) search tool.

Protein	Subunits	Reference for BLASTp
***Methanococci* and *Methanopyrus kandleri*:**		
Formate transporter	FdhC	*Methanococcus maripaludis* (Sattler et al., 2013)
Formate dehydrogenase	FdhAB	*Methanococcus maripaludis* (Wood et al., 2003)
Membrane hydrogenases	EhaABCDEFGHIJKLMNOPQ<br>EhbABCDEF(H/I)JKLMNOPQ	*Methanococcus maripaludis* (Lie et al., 2012)
F_{420}-reducing hydrogenase	FrhBGAD	*Methanococcus maripaludis* (Hendrickson & Leigh, 2008)
Heterodisulfide-reducing hydrogenase	VhuDGAB	*Methanothermobacter marburgensis* (Kaster et al., 2011)
Methylene-H_4MPT dehydrogenase	Hmd	*Methanococcus maripaludis* (Hendrickson & Leigh, 2008)
***Thermococci*:**		
Formate transporter	FdhC	*Methanococcus maripaludis* (Sattler et al., 2013)
Formate hydrogenlyase	Fhl (17 subunits)	*Thermococcus onnurineus* (Moon et al., 2012)
NAD(P)H:formate dehydrogenase	Nfd (5 subunits)	*Thermococcus onnurineus* (Yang et al., 2022)
Membrane hydrogenase	MbhABCDEFGHIIJKLMN	*Pyrococcus furiosus* (Sapra et al., 2003)
Sulfhydrogenase	ShBGDA	*Pyrococcus furiosus* (van Haaster et al., 2008)
F_{420}-reducing-like hydrogenase	FrhAGB	*Thermococcus onnurineus* (Jung et al., 2020)
Carbon monoxide dehydrogenase	Codh	*Thermococcus onnurineus* (Moon et al., 2012)
**Archaeal purine biosynthesis:**		
Formylglycinamide-ribose-5-phosphate synthetase	PurT	Brown et al. (2011)
Forminidoimidazole carboxamide-ribose-5-phosphate synthetase	PurP	*Methanocaldococcus jannaschii* (Ownby et al., 2005; Brown et al., 2011)
